# Structural Insights into ATP-Sensitive Potassium Channel Mechanics: A Role of Intrinsically Disordered Regions

**DOI:** 10.1021/acs.jcim.2c01196

**Published:** 2023-02-06

**Authors:** Katarzyna Walczewska-Szewc, Wiesław Nowak

**Affiliations:** Institute of Physics, Faculty of Physics, Astronomy and Informatics, Nicolaus Copernicus University in Toruń, ul. Grudziądzka 5, 87-100 Toruń, Poland

## Abstract

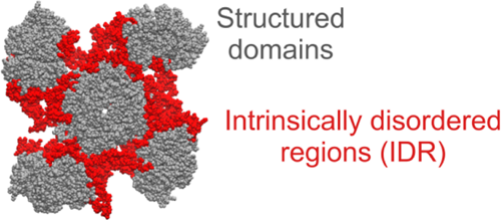

Commonly used techniques, such as CryoEM or X-ray, are not able to capture the structural reorganizations of disordered regions of proteins (IDR); therefore, it is difficult to assess their functions in proteins based exclusively on experiments. To fill this gap, we used computational molecular dynamics (MD) simulation methods to capture IDR dynamics and trace biological function-related interactions in the Kir6.2/SUR1 potassium channel. This ATP-sensitive octameric complex, one of the critical elements in the insulin secretion process in human pancreatic β-cells, has four to five large, disordered fragments. Using unique MD simulations of the full Kir6.2/SUR1 channel complex, we present an in-depth analysis of the dynamics of the disordered regions and discuss the possible functions they could have in this system. Our MD results confirmed the crucial role of the N-terminus of the Kir6.2 fragment and the L0-loop of the SUR1 protein in the transfer of mechanical signals between domains that trigger insulin release. Moreover, we show that the presence of IDRs affects natural ligand binding. Our research takes us one step further toward understanding the action of this vital complex.

## 1 Introduction

Over decades, it has been known that the paradigm linking a protein’s biological function to its well-defined three-dimensional structure is not always fulfilled. Most of well-known proteins still fold quite precisely, adopting their function-related structures. However, a significant proportion of proteins, or protein fragments, escape this rule and perform important tasks in the organism without exhibiting a defined secondary structure^1,2^

Intrinsically disordered proteins (IDPs) or intrinsically disordered protein regions (IDRs) can adopt a whole ensemble of conformations and act while remaining unstructured. Analyses show that up to 67% of eukaryotic proteins contain at least one disordered region.^1^ Those include the ATP-sensitive potassium (KATP) channels discussed in our paper.

Human KATPs are octameric transmembrane (TM) complexes commonly present in smooth muscles, cardiac myocytes, brain, and pancreatic beta-cells.^3^ Pancreatic KATP channels, main objects studied here, open or close in response to physiological changes in the ratio of ATP to ADP present in the cytosol. Their particular conformational states affect the concentrations of potassium ions on both sides of the membrane and through the resulting change in the membrane potential Kir6.2/Sur1 channels mediate insulin secretion from pancreatic beta-cells. Dysfunctions of the channel caused by mutations may lead to neonatal diabetes^4^ or congenital hyperinsulinism.^5^

**Figure 1:**
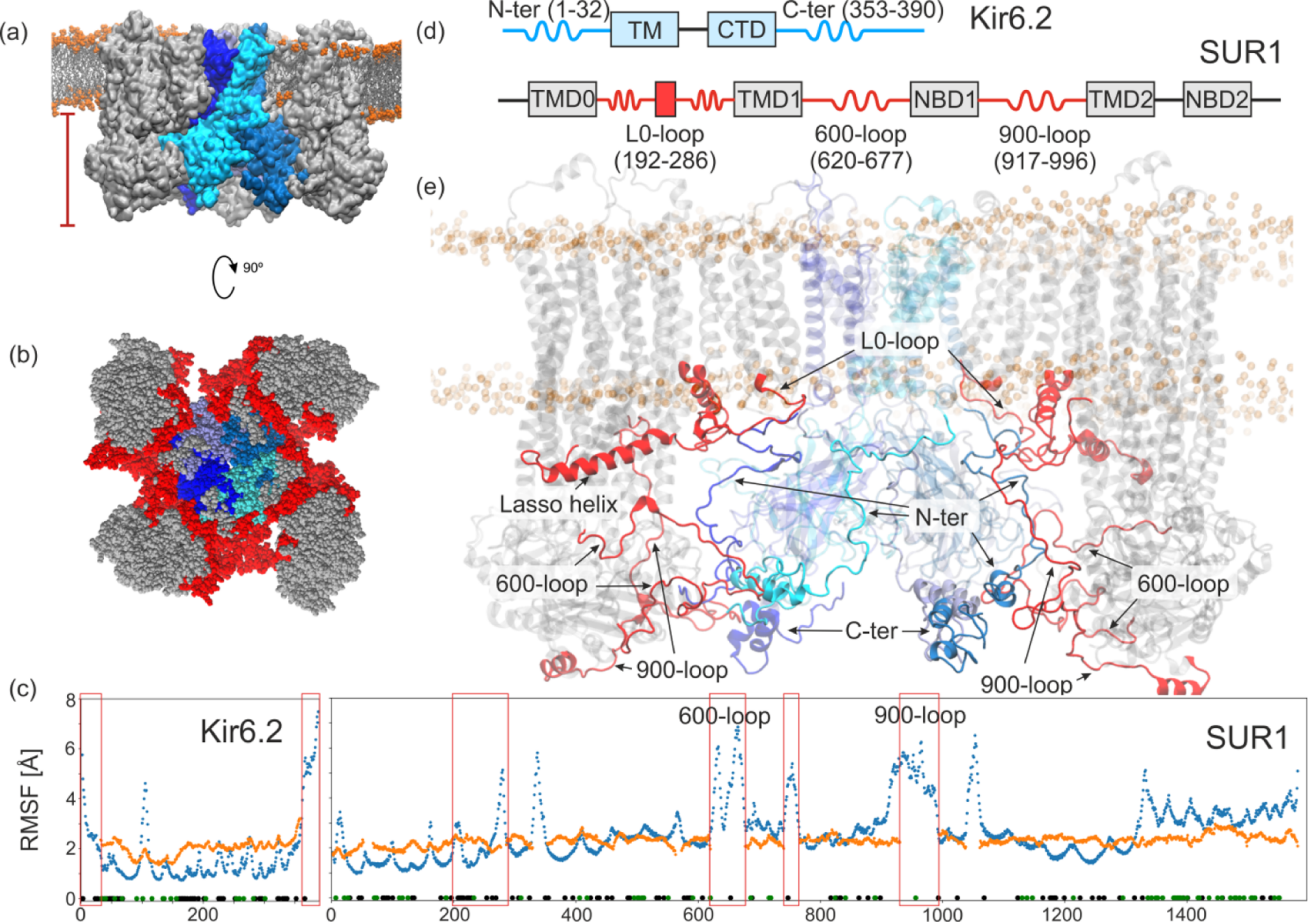
Simulated system of KATP octamer (a). SUR1 is in gray, Kir6.2 subunits are in shades of blue, and lipid heads phosphorus atoms are orange. The cytoplasmic part of the KATP complex with the inter-domain interface formed purely with IDRs (red) (b). Root-mean-square-fluctuation (RMSF) of Kir6.2 and SUR1 structures during MD simulation (blue) compared to an experimental temperature factor (orange)^19^ (c). The domain structure (d) and the cross-section of the channel (e). IDRs (N-ter, C-ter, 600-loop, and 900-loop) and partially disordered region (L0-loop) are indicated in red and blue for SUR1 and Kir6.2, respectively.

IDRs are commonly found in TM proteins, regulating their permeation and recruitment of downstream interaction partners.^6,7^ Recent CryoEM studies indicate that pancreatic human KATPs have at least five regions which may be classified as IDRs. One may expect those regions to play a role in the KATP function. However, they were not analyzed so far. Particularly, this is the case for 32 amino acids long, unstructured N-terminus of the Kir6.2 protein (N-ter), whose relevance in the channel function was indicated by many experimental studies.^8−10^ Besides, a glutamate-rich linker of SUR was suggested recently by Sung et al.^11^ as a component of a conformational pathway toward vascular KATP channel activation. In multi-domain systems, IDRs often mediate the propagation of a perturbation arising at a specific location in one domain to a distal location.^2^ Therefore, given that the cytosolic part’s interface between KATP domains is built mainly from IDRs (Figure 1b), this issue is worth investigating.

Experiments and various simulation methods allow us to study the structure and evolution of IDP and IDR. In such experiments, small angle X-ray scattering,^12^ nuclear magnetic resonance^13^ (NMR), and Förster resonance energy transfer^14^ (FRET) methods are typically used. Simulations often complement the abovementioned techniques to delineate IDP ensembles that the experimental parameters can describe. In the case of numerical modeling methods, these are mainly different types of molecular dynamics (MD) simulations but also, for example, methods based on Markov chains,^15^ rotamer libraries,^16^ and Dynamic Meccano approaches.^17^ In this paper, using novel, based on CryoEM data, models of the human KATP channel and the first all-atom MD simulations of the complete KATP system on microsecond scales, we explore the conformational space of IDRs in KATP. We monitor and show how IDR structures evolve over time. We check whether they adopt any transient, well defined, conformations or yet they remain completely disordered on the present simulation timescale. We also discuss possible roles of IDR as mediators of interactions between KATP subunits. We investigate how IDRs may affect the dynamics of the protein in the form we know so far. Such knowledge will help us to understand better how KATP, so critical for physiology, works.

## 2 Results and Discussion

### 2.1 Where IDRs in KATP are Located? Overview of Major IDRs in KATP

The KATP complex consists of eight units (see Figure 1a,b). Four of them, named Kir6.2, form the inner part lining the inward-rectifier potassium channel’s pore. Outside this inner core are located four sulfonylurea receptors—SUR1 units. They have an important regulatory function. SUR1 mediates the activation effect of Mg-ADP and affects the pharmacological profile of the channel.^18^ The structure of the main channel components is well known.^19−21^ CryoEM maps show two major conformations of human KATP: propeller and quatrefoil which differ in the position of the SUR1 subunits.^19^ The propeller conformation is thought to be dominant in pancreatic KATP in a closed state.^21,22^ Both Kir6.2 and SUR1 subunits consist of TM and cytosolic parts. In the case of Kir6.2 subunits, there are two TM helices and one cytoplasmic domain (CTD). SUR1 has the structure consistent with other ABC transporters architecture—it is composed of two membrane-spanning domains (TMD1 and TMD2), two cytosolic nucleotide-binding domains (NBD1 and NBD2), and an additional transmembrane domain (TMD0) functioning as an interface between Kir6.2 and SUR1. In the whole KATP complex, 15% of the structure is not defined in the available CryoEM data.^19^ These include 17% of Kir6.2 and 14% of SUR1. Most of such regions are intrinsically disordered (IDR-intrinsically disordered region, see Figure 1c–e). It is assumed that the dynamical nature of IDRs makes them “invisible” for structural characterization methods like X-ray crystallography or CryoEM.^23,24^

In Kir6.2, the major IDRs are N-terminus (N-ter) and C-terminus (C-ter) which partially form the CTD. In SUR1, three loops: the 600-loop connecting TMD1 to NBD1, the 900-loop connecting NBD1 to TMD2, and the 700-loop belonging to NBD1 are fully disordered in known CryoEM structures. These fragments meet the accepted definition of an IDR that is a fragment of 25 or more residues without a noticeable secondary structure.^1^ Moreover, polar (S, T, Q, D, E, R, K, and H) and structure-breaking (G,P) residues are more common in such regions and hydrophobic, aromatic, and cysteine and asparagine residues are rare. Here, we have included fragments located on the inner side of the cell membrane.

We also decided to analyze the L0-loop with Lasso helix motif connecting TMD0 to TMD1 which is partially disordered (although nearly fully covered in CryoEM data) because of the potentially important role, this fragment plays in the KATP function.

Below we provide a short description of each selected, disordered fragment.

The N-terminal end of Kir6.2 (N-ter) is recently a subject of vigorous research, as it was postulated that this flexible polypeptide chain has a major factor in controlling KATP opening/closing processes.^9,10^ Our previous studies have confirmed that N-ter from Kir6.2 can reach and explore the sulfonylurea drug binding region in the middle of SUR1 and can keep SUR1 in the inward open form.^25^ The disordered N-ter region from aa 1 to 32 is unsolved in “early” structures. In some cases, for example, 6C3P, 6C3O, and 5YKF, it is because structures have been resolved using the C-terminal of SUR1 and N-terminal of the Kir6.2 fusion construct. However, recent structures from Chen and Shyng groups show fair density of N-ter in the drug-binding cavity of SUR1 being in the inward open state.^8,10^ The possible positions and localization of the N-ter when SUR1 is in the outward open conformation remain unknown. Numerous experimental data indicate the importance of N-ter in proper KATP functioning.^10,26,27^ Moreover, numerous mutations can disrupt its function, for example: L2P, S3C, A28-R32 deletion, F33I, R34C, and F35L cause NDM.^28,29^

The C-terminal fragment of Kir6.2 (C-ter) from aa 353 to 390, absent in available structures, contains an endoplasmic reticulum retention motif (RKR) that prevents the cell’s surface expression of Kir6.2 in the absence of the SUR1 subunit.^30^ The RKR motif is in the region aa 369–371 and it is masked when SUR1 is present. Truncation of the gene encoding the last 26–36 amino acids of Kir6.2 causes expression of channels composed of Kir6.2 only.^31^ The mutation of glycine 366 lying near the RKR motif to the much bulkier tryptophan causes NDM.^32^

The so-called lasso motif located between TMD0 and TMD1 (aa 192–286) (L0-loop) draws attention primarily by the abundance of pathogenic mutations (NDM related) occurring in this region.^29^ Most of those mutations affect a fragment without a clear secondary structure—the N-terminal part of the L0-loop: I196N, P207S, E208K, D209N/E, Q211K, D212I/N/E/Y, L213R/P(DEND), G214R (MODY), V215I, and R216C. In another protein of the ABCC family-CFTR, this motif provides a proper conformational stability of the core ABC structure by association with the membrane.^33^ In KATP, the L0-loop may have an additional function. We know that the N-terminal L0-loop residues (199–214) contribute to the binding site of ATP in Kir6.2 (the so-called ABLOS-“ATP binding loop on SUR”).^21^ The ATP binding site partially overlaps with the PIP2 (phosphatidylinositol 4,5-bisphosphate) binding site.^34^ PIP2 is a minor phospholipid from the inner leaflet of the membrane, which binding promotes the open structure of Kir6.2.^35^ By the fact that the L0-loop directly connects the ABC core to an ATP/PIP2 binding site relevant in the opening-closing of the channel pore, the L0-loop likely is one of the factors carrying the dimerization information of SUR1 domains into the channel pore.^19^

The spatial arrangement of the other SUR1 loops, that is, 600-loop, 700-loop, and 900-loop, places them in a position where they can significantly impact the dynamics of the N-ter and ABC domain of SUR1.

An un-modeled region extending from aa 620 to 677 of the SUR1 protein (600-loop) links the TMD1 and NBD1 domains. This fragment also contains the endoplasmic reticulum retention motif (RKR aa 648–650), preventing protein expression without KATP assembly. Similar to C-ter, mutations near the RKR motif (R653Q) lead to transient neonatal diabetes.^36^ KATP channels found in different human tissues contain different Kir/SUR subunit combinations. In pancreatic beta cells, these are Kir6.2 and SUR1, but in vascular smooth muscles, the latter is replaced by SUR2B. The L1 linker, a 600-loop analogue in SUR2B, has a phosphorylation site. Phosphorylation of the L1 linker activates the cardiac KATP channel.^37^ No such activity appears in SUR1. Additionally, L1 in cardiac SUR2A has a heme-binding motif, absent in SUR1.^38^ Thus, it could be that the additional (beyond RKR) functionality of the 600-loop in SUR1 has disappeared during evolution.

700-loop is a part of the NBD1 domain. It includes residues from 740 to 766. There is no information about possible posttranslational modifications of this region in the available literature. However, it has been reported that the nonsense mutation E747X causes neonatal diabetes.^39^

The undefined fragment ranging from aa 917 to 996 (900-loop) is intriguing as it contains the region especially rich in glutamic acid residues (the ED domain, following Sung et al.^11^). Glutamic acid is the second of the most common disorder-promoting residues.^40^ Moreover, such a motif of seven glutamic acids in a row (aa 969–975) is conserved in all SUR-type proteins (SUR1, SUR2A, and SUR2B) in all organisms. Interestingly, this motif does not appear in other ABC transporters, raising the possibility that it may be closely associated with the functioning of KATP channels. Indeed, the tripartite interaction between 900-loop, SUR2B, and Kir6.1 has been recently reported for smooth muscle KATP channels.^11^ It is known that glutamic acid/aspartic acid clusters coordinate various metal ions binding or destabilize the structure leading to weakening the ion binding capacity.^40^ A reported mutation in this region causing neonatal diabetes that disrupts the channel function is R993C.^36^

### 2.2 Whole KATP System MD Simulations

**Figure 2:**
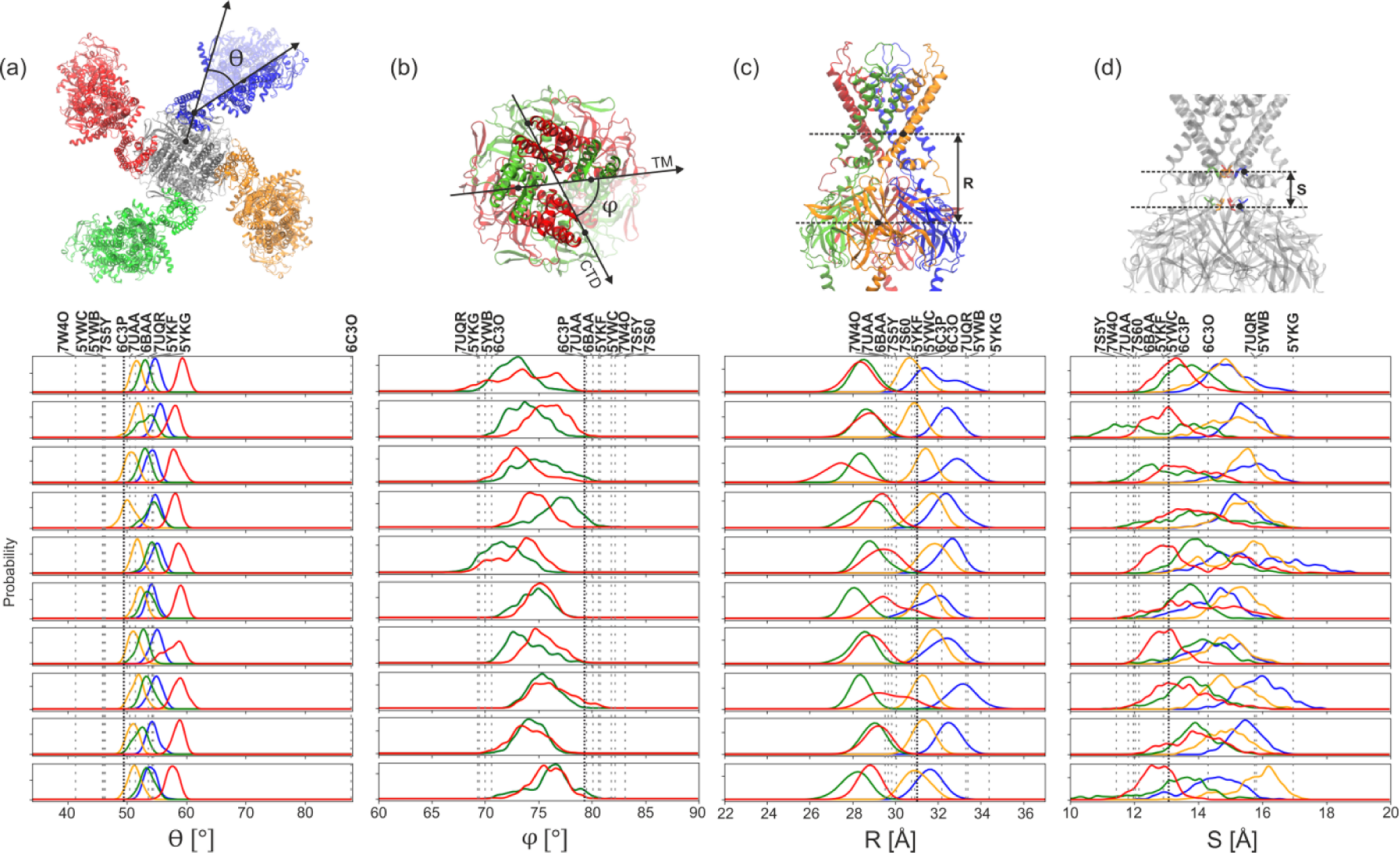
Changes in system geometry monitored during MD simulation. Propeller angle between a vector joining TM (residues 70–170) part of Kir6.2 with TMD0 of SUR1 (1–192) and a vector joining TMD0 with the center of mass of TMD1 (295–620) and TMD2 (1007–1320) domains for each chain (a); rotation angle of the CTD domain (170–350) in relation to the TM part of Kir6.2 for two chain pairs (b); distances between CTD center of mass and HBC (F168) for each chain (c); and distance between center of mass of residue I298 from CTD and HBC for each chain separately (d). Vertical lines show values calculated for the CryoEM structures.

The model used in our simulations is based on the 6C3P CryoEM structure.^19^ In Figure 2, we present a series of structural parameters, ordering a selection of the available structures. Most of them were characterized in a recent review by Driggers and Shyng.^41^ We have included structures that differ in terms of the dimerization of the NBD domains, the openness of the channel pore, the rotation of the SUR1 part (propeller and quatrefoil form), and the rotation and the positioning of the CTD domain relative to the TM part. The 6C3P structure represents a KATP channel in the propeller conformation (Figure 2a—propeller angle) with activated, dimerized NBD SUR1 (outward facing) domains. Although this is one of the signatures of the open channel state, the channel pore remains closed. The characteristic rotation angle of the CTD domain in Kir6.2 (Figure 2b—CTD rotation angle) indicates a tense “T” state according to Wu et al.^21^ 2018 nomenclature. In addition, the distance of the CTD domain from the amino acids forming the so-called helix bundle crossing (HBC) (Figure 2c,d) places the structure in a CTD-“up” state (according to Sung et al.^8^ 2022 nomenclature). According to the leading hypothesis, the CTD-up conformation enables ligand modulation.^41^ The 6C3P structure, therefore, represents a rational intermediate point among the available structures.

In our relatively long simulation time (10 repetitions of 0.5 μs each), the model of the system as a whole remained relatively stable. The characteristic propeller shape was retained; however, there is noticeable anisotropy among SUR1 chains (see Figure 2a). The ABC domains of SUR1 remained in the outward open conformation throughout the simulation, with NBD1 and NBD2 domains remaining close to each other and with MgADP bound at both consensus and degenerate binding sites (NBD1 and NBD2). Fluctuations of the whole system are shown in Supporting Information in Figure S2. Selected ID regions had fluctuations reaching 5–12 Å above the general fluctuation level. The separate root-mean-square deviation (rmsd) of the fragments studied for each of the simulations performed (10 repeats × 4 protein chains) is shown in Supporting Information, Figure S3. The conformational dynamics of the CTD domain of Kir6.2 also shows some anisotropy for the different chains (see Figure 2b–d). However, these changes occur within limits observed by the available structures (vertical dashed lines). In Figure 2c,d, the vertical dashed lines show distances between CTD center of mass and HBC and residue I298 from CTD and HBC in experimentally determined structures. Those values are averaged over the four chains although the discrepancies in the distances of the individual chains range up to 3 Å depending on the structure.

**Figure 3:**
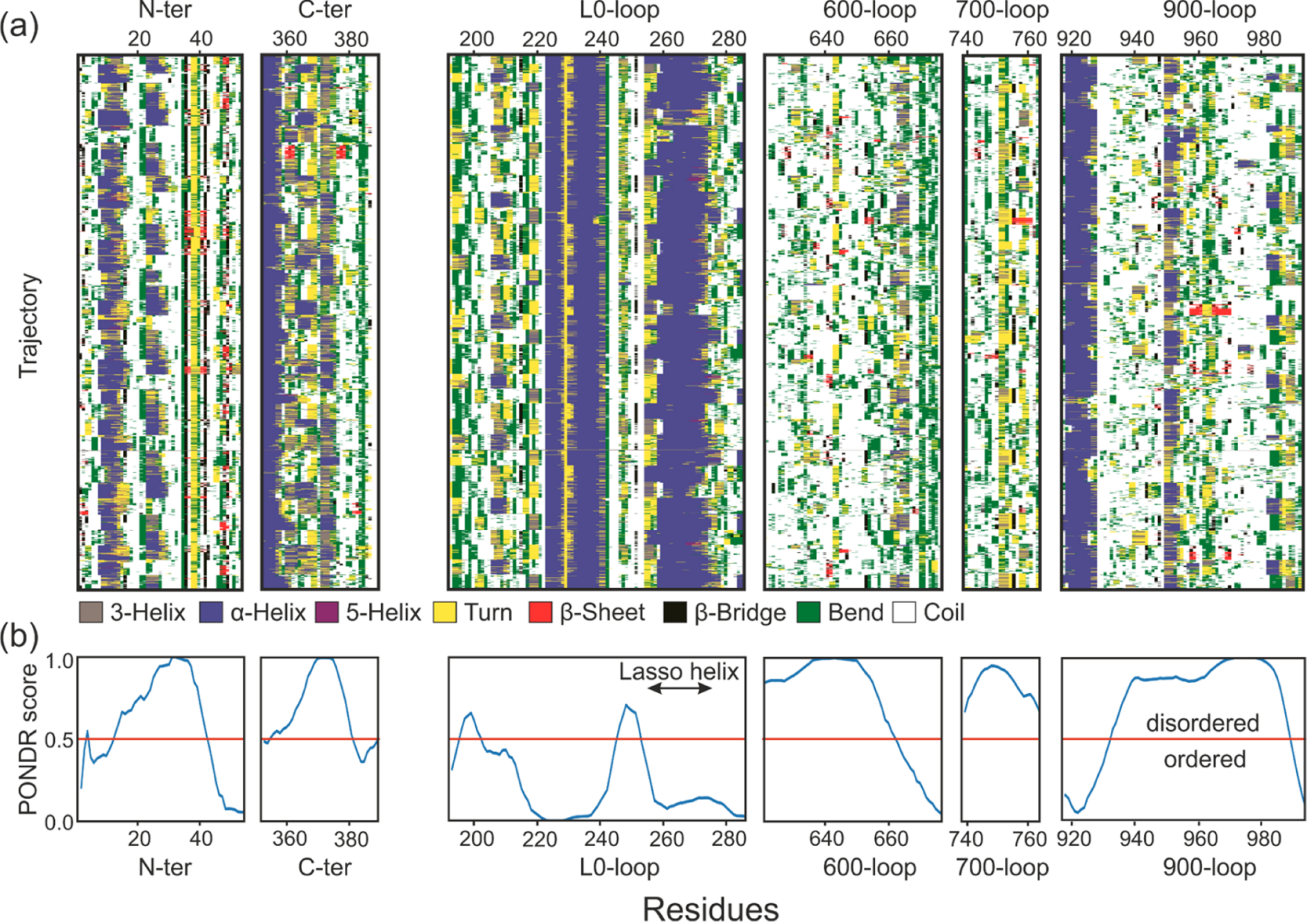
Time evolution of the secondary structure of IDRs as calculated by DSSP (a). The *x*-axis displays data collected during 0.5 μs for each chain for nine trajectories. Disorder in selected regions (b). The PONDR prediction tools were used to determine the disorder score. Any value above 0.5 indicates intrinsic disorder.

In Figure 3a, we show the changes in the secondary structure of the disordered parts of the system during the simulation. In the case of the N-ter, we see a propensity for the α-helix formation for residues 8–16 and 20–30, but these are not stable structures. Above residue 37, the stability of the structure is affected by several interactions with CTD. For C-ter, the disordered fragment starts around residue 360 that ends the last ordered fragment of the CTD. Some fragments, such as aa 365–370, can form temporary α-helices, but those are short-lived phenomena. The L0-loop contains two helices, found in CryoEM. As expected, these fragments maintain the helical structure throughout the simulation. Among those helices, the structure is disordered and, apart from a slight tendency toward helicity for a fragment around residue 210, no significant regularities can be seen in the diagram. The other SUR1 loop analyzed, the 600-loop, shows the greatest disorder. The sporadic structures appearing are very short-lived and rather do not indicate specific interactions with the environment. In the last analyzed loop of SUR1, the 900-loop, the disordered fragment starts from residue 930, at the point where the α-helix belonging to NBD1 ends. In the diagram, we see the final fragment of this helix remaining in a stable form throughout the simulation. A further disordered fragment shows some tendency to helicity in the vicinity of residues 942 and 985.

The disorder score calculated from the amino acid sequence reflects the dynamics of the fragments during the simulation. In particular, for N-ter, L0-loop, and 900-loop, where we also included fragments of ordered regions of the protein in the analysis.

### 2.3 Conformational Space of Unbound N-ter

Experimental studies^8−10,26,42^ and our previous numerical studies^25^ indicate that Kir6.2 N-ter has an important function in the communication between SUR1 and Kir6.2. An extended fragment protruding from the channel shaft and docked in the SU pocket of SUR1 allows, from one side, to keep the CTD domains of Kir6.2 activated and, from the other side, to stabilize the inward open conformation of the ABC core. The presence of Kir N-ter in the interior of the ABC-core also affects the possible binding of sulfonylureas.^43^ Moreover, the C-terminal part of N-ter is known to participate in the ATP binding.^20,44^ However, we still do not have any information on what happens with N-ter when it is not bound inside the SUR1 niche—that is, when the SUR1 conformation changes to outward open.

The performed MD simulations gave us information about the evolution of this fragment over time. Ten repetitions of a 0.5 μs simulation of a system containing four copies of the Kir protein provides us with a total of 20 μs sampling of the N-ter conformational space itself. Despite smaller than expected rmsd values oscillating between 2 and 6 Å (see Supporting Information, Figure S3), the fragment does not appear to have a well-defined “parking place” in the overall KATP structure. N-ter moves relatively freely in the confined space between the CTD and the ABC-core (Supporting Information, Figure S4) without entering into more permanent interactions with any part of the system—none of the traced contacts between residues forms for more than 50% of the simulation time (see Figure 3c). The most frequent interactions (reaching 40%) are salt bridges formed between charged residues in the middle and end part of N-ter and residues from NBD1 of SUR1 (E19-K795 and R27-E784) and CTD of the same (E19-H276) or neighboring Kir6.2 (R25-E321). Interactions of N-terminal parts of N-ter with disordered 900-loop (R4-T949 and K5-T949) are also quite frequent at the 30% level. This is, in our opinion, an interesting observation. The KATP system has such architecture and aa composition in this region that any permanent binding of N-ter is of low probability. It means that flexible N-ter is “ready” all the time to sneak into SUR1 and to stabilize open forms of SUR1 (channel is closed). Perhaps, such lack of permanent “docking positions” for N-ter facilities its role as a conformational lock (or “selector”). Tightly bound N-ter might be useless in this task or its efficiency would be lower.

### 2.4 L0-loop

The L0-loop is not a strictly disordered region. It has no defined structure in only one of the discussed conformations of the KATP—a quatrefoil state. Thus, it is almost completely well-determined by the available CryoEM structures in the propeller conformation. However, this region still has some flexibility, especially in its N-terminal side, whose amino-acid composition favors a lack of order (see PONDR score, Figure 3b). Although the space available for L0-loop movement is severely limited by the presence of other subunits of the system, the rmsd of the fragment in our simulations is of the order of 6 Å (Supporting Information, Figure S3), implying substantial conformational freedom.

The L0-loop behavior is determined by stable interactions between the short helices comprising L0 and the TMD1 domain. These interactions occur in more than 80% of the simulations (see Figure 5a,b). Of particular interest is that L0-loop directly interacts with the residues responsible for binding sulfonylureas (anti-diabetic drugs used to close the KATP channel), namely, R1245 and E1246.^43^

**Figure 4:**
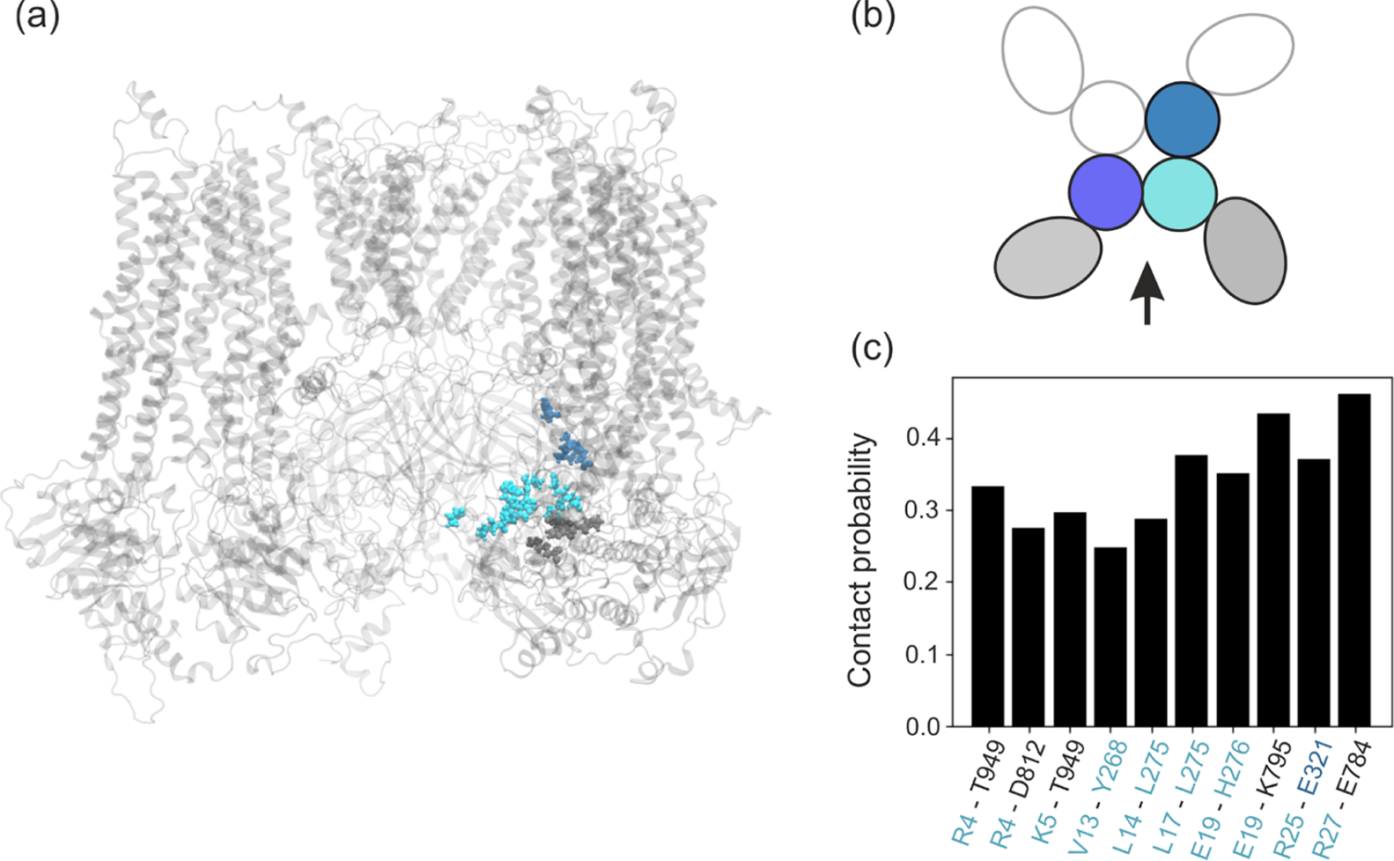
Spatial distribution of the residues most often in contact with N-ter assigned by color to the parts of the system to which they belong (a). Schematic, color-coded, and representation of the KATP system. Colors are assigned to each part throughout the paper: shades of blue—individual Kir6.2 chains, gray and black -SUR1 (b). Frequency of close contact between N-ter residues and the rest of KATP (c).

**Figure 5:**
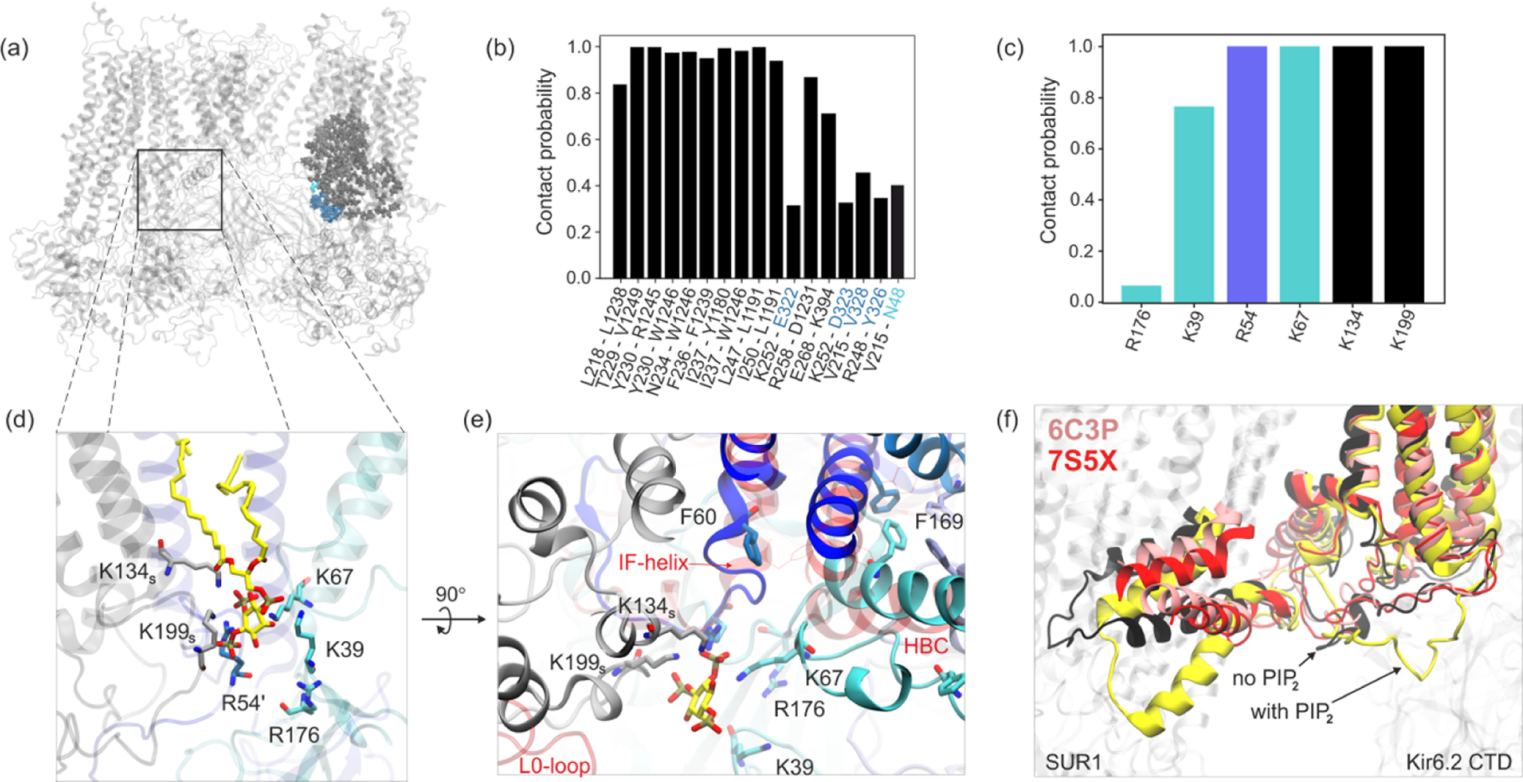
Spatial distribution of the residues most often in contact with the L0-loop (a). Frequency of close contact between L0-loop residues and the rest of KATP (b). Contribution of Kir6.2 and SUR1 residues to PIP2 binding (c). Close-up view of the Kir6.2 PIP2 binding site (d). Location of the PIP2 binding site with respect to the pore and HBC (particularly F169) and side helix (residue F60)—top view (e). The position of such regions in KATP in the open conformation is shown in red. Comparison of L0-loop positions in the presence (yellow) and absence of PIP2 (black) with experimental data—6C3P (pink) and 7S5X (open Kir6.2, red) CryoEM structures (f).

In addition, experimental data indicate a direct involvement of the N-terminal part of L0-loop in ATP binding at the inhibitory site in Kir6.2. The CryoEM structure of Ding et al.^9^ shows that the side chain of K205 in SUR1 coordinates the β- and γ-phosphates of ATP. Therefore, these authors named residues 199–214 of SUR1 the “ATP binding loop on SUR” (ABLOS). Such a motif is highly conserved from fish to humans,^9^ and as mentioned before, several reported mutations in this region lead to NDM.

Our channel model is constructed based on the 6C3P structure^19^ (see Section 2.2), which originally has four ATP at the inhibitory sites of Kir6.2. However, in our model, ATP is absent. Thus, we cannot explicitly show how the L0-loop participates in ATP binding. We can show that the L0-loop remains in direct contact (almost 40% of the simulation time) with the CTD domain of the neighboring Kir6.2 and N48, which is an extension of the N-ter region of the adjacent Kir6.2. The last residue N48, participates directly in ATP binding.

The absence of ATP at the inhibitory site of Kir6.2 and the presence of PIP2 in the lower membrane leaflet promotes PIP2 binding to the KATP system. In our model, of the four available binding sites, in two, PIP2 is stably bound to the channel, in one, it is occasionally bound, and one site was vacant throughout all simulations (see the Supporting Information). Our characterization of the PIP2 binding site is in agreement with recent numerical results^34^ (see Figure 5c,d). It mainly consists of residues K39 and K67, and R54 from the neighboring Kir6.2 unit. The arginine R176, due to the rather strict definition of close contact adopted by us, is not directly involved in PIP2 binding, although it remains nearby. The PIP2 binding site is co-formed by residues belonging to SUR1. The recent work of Sung et al.^8^ confirms that K134 from the TMD0 domain of SUR1 can interact with PIP2. This is consistent with our studies, which indicate that when PIP2 is present at the binding site, the close contact between PIP2 and K134 occurs in nearly 100% of simulation time. Additionally, the N-terminal L0-loop (ABLOS) lysine K199 is one of the five positively charged amino acids that form the optimal environment for PIP2 binding.

Although PIP2 binding favors the open KATP conformation, comparing our model after 500 ns of simulation with the open channel structure from CryoEM (PDB ID: 7S60),^45^ we can see some differences (see Figure 5e). The transparent red structure corresponds to the 7S60 structure. We can see some discrepancies in the position of HBC (in particular, in the position of F168), indicating that the pore is closed. The interfacial helix (IF) is slightly offset in our case, and the critical interaction between F60 and F168 described by Zhao and MacKinnon in the open KATP form does not occur.^45^ Additionally, the L0-loop in our model moves closer to Kir6.2, allowing a direct interaction with PIP2, whereas in 7S60, the L0-loop is retracted. The difference in the L0-loop conformation results from the presence of PIP2 in our model. As can be seen in Figure 4f, in the absence of PIP2 in some chains in our model (black structure), the position of L0 is already consistent with the positions obtained experimentally for 7S60, 7S5X(45) (red structure), and 6C3P(19) (pink structure).

### 2.5 Intrinsically Disordered Loops of SUR1 as a Part of Hypothetical Molecular Bearing

**Figure 6:**
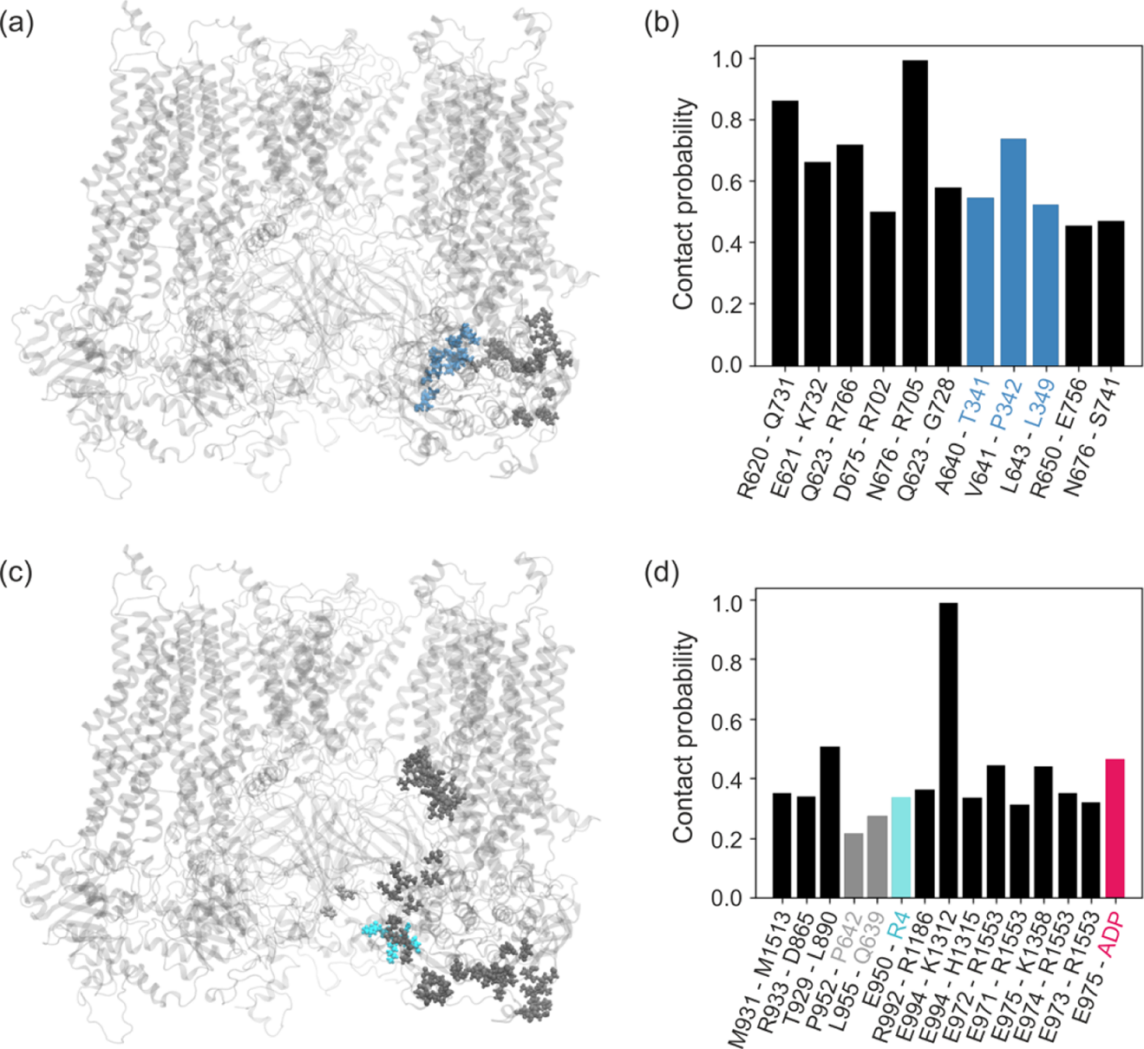
Spatial distribution of the residues being most often in contact with 600-loop (a) and 900-loop (c). Frequency of close contact between Kir6.2 and SUR1 residues and 600-loop (b) and 900-loop (b,d).

The flexible loops connecting NBD1 to the TM domain are the most extended fully disordered fragments in KATP. Sterically, they are the only fragments of SUR1 subunits that can directly interact with adjacent ones. Therefore, one of the proposed functions performed by these fragments is to stabilize the characteristic propeller shape of the KATP complex. Indeed, contacts between neighboring SUR1s subunits through 600-loop and 900-loop loops occur in 20% of the simulation time (see Figure 6d). Another KATP structure observed in CryoEM is quatrefoil conformation where SUR1 units are rotated counter-clockwise.^19^ Nevertheless, because we have not considered the less frequent quatrefoil conformation of pancreatic KATP or the conversion between these two forms, such a hypothesis still needs to be confirmed. On the other hand, 600-loop and 900-loop provide a necessary flexibility (or conformational freedom) to all four SUR1 domains: the 600-loop connects TMD1 to NBD1, the 900-loop connects NBD1 to TMD2. At the same time, the presence of those ID loops probably reduces friction between Kir6.2 and SUR1 subunits during open/close KATP cycling. Thus, a critical analysis of dynamics of this IDR is presented here.

In SUR proteins (i.e., SUR1, SUR2A, and SUR2B), the 600-loop is longer than in other proteins of the ABCC family (e.g., CFTR and MRP1 with known atomic structure), which may indicate some particular function this loop has in KATP channels. We have already mentioned that SUR1 has an endoplasmic reticulum retention motif, ensuring co-expression of SUR1 with Kir6.2. In SUR2B, on the other hand, it has been shown that 600-loop’s phosphorylation regulates channel activity^23,46^—disruption of the loop interactions with NBD1 leads to increased affinity of NBD1 for MgATP. NMR studies of Sooklal et al.^23^ on an isolated 600-loop—NBD1 system from rodent SUR2B show that the 600-loop possesses a residual secondary structure that is disrupted with phosphorylation. Such disruption leads to changes in the interaction pattern of the phosphorylated 600-loop with NBD1.

Despite the similar length of the 600-loop in SUR1, neither available experimental data nor numerical predictions indicate the existence of phosphorylation sites here (see UniprotKB database). Furthermore, if we assume some similarity in the functioning of the 600-loop with SUR1 and SUR2B, we would expect to observe some temporary structures in the un-phosphorylated state, stabilizing the 600-loop interactions with NBD1. The MD data obtained for 600-loop of SUR1 do not show the formation of such transient structures (see Figure 3a). Nevertheless, the close contacts frequency analysis reveals a relatively stable interaction of 600-loop with NBD1 residues (Figure 6a,b). Despite the large conformational freedom displayed by the 600-loop (Supporting Information, Figure S4), some of the resulting interactions are very stable, for example, the pair of N676-R705 residues remains nearby almost throughout the simulation (see Figure 6b). Also, we observe frequent proximity for the residues R620-Q731, a particularly important feature as Q731 interacts directly with W688, which is a part of the ATP binding site in NBD1 (degenerate site). Additionally, the 600-loop residues interact with CTD of Kir6.2 adjacent to the unit interacting with SUR1 through the TMD0 domain. High flexibility of this CTD neighborhood facilitates functional motions of Kir6.2 CTD (molecular bearing). Due to the proximity in space of the 600-loop and 700-loop, interactions between them are also frequent.

The second fragment connecting NBD1 to the transmembrane part (TMD2) is the longest disordered fragment of SUR1. So far, little is known about its function. In other proteins of the ABCC family, that is, CFTR and MDR1, this fragment is even longer (200 and 90 amino acids, respectively). In the former case, it forms a separate R domain, which regulates the protein’s function. Additionally, in both cases, it is phosphorylated which may modify the function of the fragment.

In SUR proteins, the 900-loop fragment is approximately 80 amino acids long. It does not contain experimentally confirmed phosphorylation sites, although prediction servers such as PhosphoSite suggest possible phosphorylation of serine 979 in SUR1. Due to its location at the system’s periphery, the 900-loop samples a significant volume over time, compared to other IDRs (see Supporting Information, Figure S5). The principal components analysis (PCA)^47^ allows us to distinguish three groups of the most common conformations, with different courses of the loop. There is a substantial anisotropy in the dynamics of the loop for each SUR1 subunit. Nevertheless, we can identify several hot spots in the interaction of the fragment with the rest of SUR1 (see Figure 6c,d). More than 40% probability of close contacts is shown by the pairs T929-L890 of NBD1 and E972-R1553 and E974-K1358. The first residue in the two last pairs belongs to an unusual accumulation of seven glutamic acids (ED), while the other belongs to the C-terminal and N-terminal parts of NBD2, respectively. The end of the disordered 900-loop fragment is determined by the highly stable salt bridge connecting E994 to K1312 belonging to TMD2. These residues are close to each other in the CryoEM structure 6C3P.^19^ Residues 990–996 are also involved in the interaction with the membrane. Interestingly, the 900-loop region, especially the ED domain, interacts directly with the N-ter (almost 40% probability of contact with R4), and in more than 40% of cases, it co-forms the MgADP binding site (consensus site).

**Figure 7:**
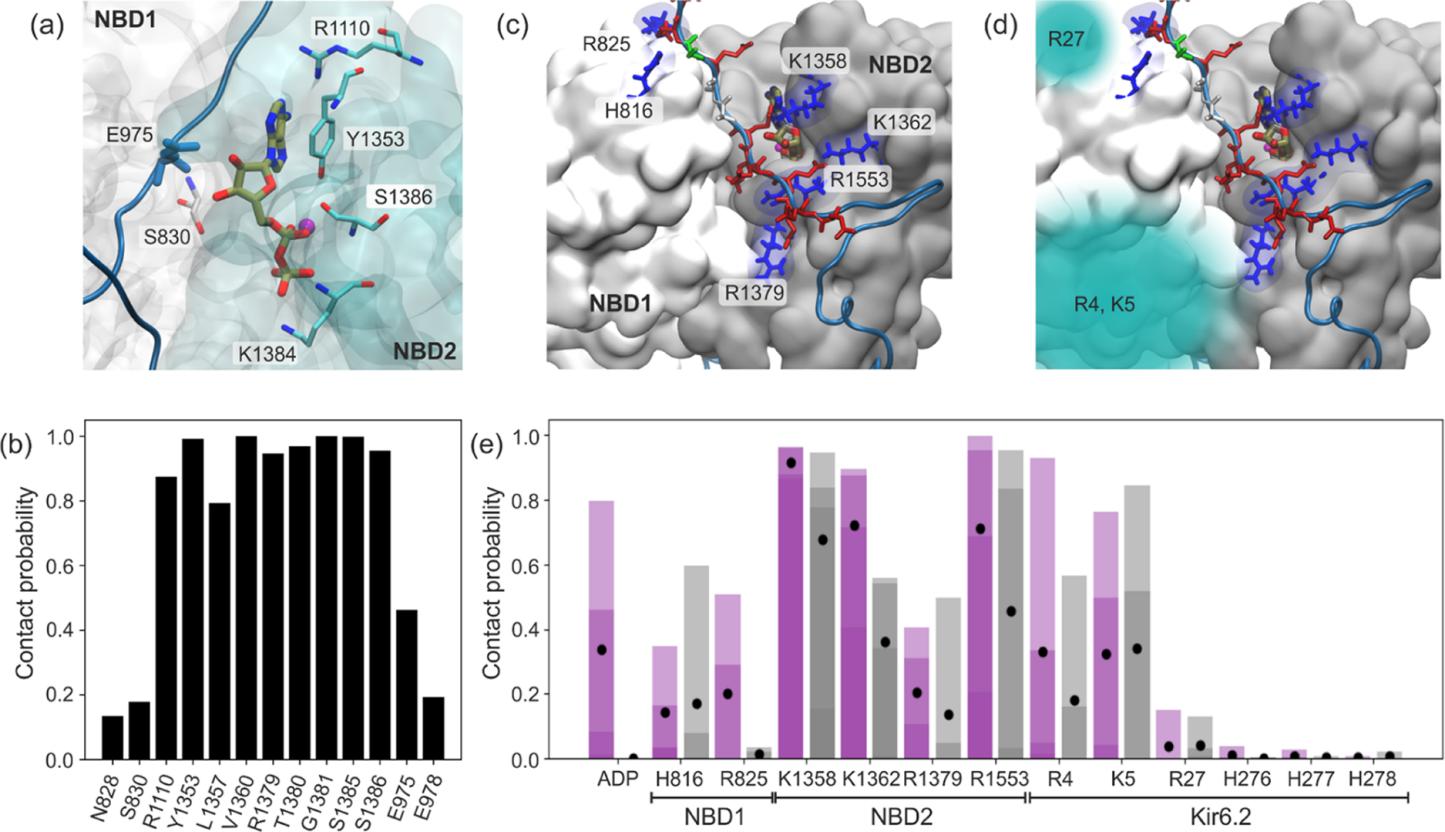
Close-up view of the consensus MgADP binding site in SUR1 NBDs (a). Frequency of close contact between MgADP and KATP residues (b). Location of positively charged anchors in NBD1 and NBD2 (blue), interacting with the ED domain (red) (c). Areas where interaction with the N-ter can occur (d). Frequency of contacts between ED domain and the “anchor” residues in two cases: ADP present in the binding pocket (magenta) and the APO model (gray) (e). Due to the high anisotropy between KATP units (chains), the contact frequencies of each chain are shown together as transparent bars. The averaged value is marked with a black dot.

In Figure 7a, the MgADP binding site is shown, together with a fragment belonging to the ED domain of the 900-loop. Additionally, we analyzed the contact frequencies of amino acids lying in the vicinity of the ligand with the MgADP ligand itself (Figure 7b). The obtained results overlap to a large extent with the experimentally determined MgADP binding site for SUR1 in the outward open conformation,^20^ that is, R1110, Y1353, S1386, and K1384, make significant contributions. In our simulation, this set is augmented by V1360, R1379, and T1380 from NBD2. S830 belonging to NBD1, a part of the experimentally determined MgADP binding site, shows a contact probability of 20% due to our strict definition of “close contact” (see Methods). The presence of residues from the glutamic acid-rich part of the 900-loop directly in the MgADP binding site suggests that this region may have arisen during evolution (and is conserved) specifically to affect MgADP binding. Indeed, Sung et al. showed MgADP dependent interaction among 900-loop, Kir6.1, and SUR2B in smooth muscle KATP channels in the quatrefoil conformation.^11^ They propose a model in which the glutamic-acid-rich part of 900-loop functions as a gatekeeper to prevent unregulated channel activation in the absence of MgADP. In pancreatic KATP channels, the propeller form appears to be the dominant conformation. Recent studies by Shyng’s group on pancreatic channels^8^ show that the 900-loop, particularly the residues near S988, comes into a close contact with the proximal part of N-ter (P24 Y26 R27) when the N-ter is wedged between the TMD1 and TMD2 domains of SUR1. Such contacts are disrupted when the CTD domain on Kir6.2 is in the “down” conformation.^8^ In our model, CTD conformation is fluctuating between the “up” and “down” state (see Figure 2a–d) and the N-ter is placed outside the SUR1 cavity. Nevertheless, we can still see occasional interactions between the C-terminal part of the 900-loop and the residues mentioned above: R27 contacts E981 for 3% of the simulation time and L985 for 2%, P24 contacts N984 for ∼5%, and R25 contacts S989 for 3% of the simulation time.

To test how the presence of the ED domain in the ADP binding site affects ligand dynamics in pancreatic KATP, we examined its fluctuations over time. During the evolution of the system, the NBD domains forming the MgADP binding site spontaneously tighten and relax yet remain closed—the distance between the centers of mass of the NBD1-NBD2 domains varies from 28 to 36 Å, compared to 45 Å in the inward open SUR1 conformation. The tight closure of the domains limits the mobility of ADP, which could potentially lead to its dissociation. The average rmsd for ADP in the tight domain closure (the distance between COM NBDs < 29.5 Å) is 1.84 Å (0.17). For such an arrangement of domains, the interaction of the ED domain with ADP occurs sporadically.

A slight relaxation of the NBD domains (COM distance > 30.5 Å) changes the situation. Here, the ED domain has free access to the ligand and interaction between the adenosine ribose hydroxyl group and the backbone (mainly oxygen in the carboxyl group) of the E975 residue occurs very frequently (see Figure 7a,b). The frequency of this interaction depends mainly on the degree of closure of the NDB domains and shows high anisotropy for different units. Such a rather unusual interaction of negatively charged glutamic acid with ADP results mainly from the interaction of the additional carboxyl group of E976 with the neighboring lysine L1358 (see Figure 7c). The presence of the ED domain seems to have a stabilizing effect on the ADP binding in NDBs—the measured rmsd of the ligand for relaxed domains is 2.07 Å (0.11) in the absence of ED, and when the interaction with ED occurs, the rmsd drops to 1.75 Å (0.1). A smaller rmsd probably translates into a lower chance of ligand dissociation. However, this hypothesis requires further computational study.

The ED domain, naturally retaining some freedom due to its disordered nature, probably prefers this specific arrangement in order to limit ADP fluctuations, that is, it anchors itself by interacting with positively charged residues of NBD domains at the interface.

One hypothesis proposed by Sung et al. is that the ED domain is a bridge between NBDs and CTDs in the quatrefoil conformation of vascular KATP.^11^ It may be involved in relaying the signal indicating the presence of ADP (and dimerization of NBD domains) in SUR2B directly to the CTDs in Kir6.1. In the recent work about pancreatic KATP, Sung et al.^8^ proposed an additional contact between 900-loop and N-ter. We analyzed both possibilities for our model of pancreatic KATP. Despite relatively long simulation times and a decent statistical sample, the NBD domains remain dimerized. We did not observe the diffusion of MgADP from the binding sites, even though relaxation of the NBD domains sometimes leads to quite significant fluctuations of the ligand. Thus, to compare the structural dynamics of the 900 loop in the presence and absence of the ligand, we used the APO model as a reference. In such a model, the MgADP binding sites in all NBDs are unoccupied. We analyzed the frequency of close contacts between the ED domain and most probable anchors in two cases: in the presence of ADP and without, each time for four different chains (see Figure 7e). Our data show that ED interacts very little with the CTD domain, making it unlikely that the signal is transmitted in that way. The contact with the Kir6.2 part of the system occurs mainly through contacts with the N-tail. Here, the positively charged R4 and K5 contribute the most (albeit with high chain related anisotropy), and R27 occasionally interacts with ED (see Figure 7d,e). There are sporadic interactions with the histidine series in the CTD. It is important to note that the percentage of ED interactions with anchors varies with the degree of closure of the NBD domains (Supporting Information, Figure S6). Direct interactions with ADP barely occur for tightly closed domains, interactions with K1362, R1379, and R1553 become less frequent, and interactions with histidines in CTD are virtually nonexistent.

### 2.6 Suggestions of New Experiments on KATP

Now, it is tempting to suggest some experimental verifications of possible physiological roles of IDRs studied here. One option is to use mutagenesis to delete the ED domain of the 900-loop in order to check pathways of Mg-ADP binding to the NBD domain consensus site. After such a genetic modification, a FRET study, analogous to that performed by Puljung et al.,^35^ might provide new data on potassium ions’ conductivity control. Also, a functional role of the L0-loop is not fully understood yet. In the quatrefoil KATP form it is disordered, but in the propeller form, dynamical second order structures are observed. Deleting selected parts of the L0 region and measurements of electrical conductivity of KATP might help to pinpoint new processes that affect KATP function. Despite the large size of the KATP system, such experiments, similar to that chasing role of IDRs in PIEZO2 mechanoactivated ion channel,^48^ should be possible.

Another possibility comes from the quickly advancing field of NMR spectral studies of IDR in proteins.^49,50^ Here, measurements of carefully selected sets of NMR ^15^N or ^13^C spin relaxations (dipolar couplings or intramolecular paramagnetic relaxation enhancement experiments) may allow us to get experimental characteristics of possible conformational ensembles in defined regions of this system. However, current studies confronting both MD and NMR approaches are rather limited to much smaller, individual proteins.^50,51^

Our MD data show that the N-ter region of Kir6.2, being in general IDR, has some propensity toward α-helix formation (50% of trajectories time). Such a helix makes the N-ter chain slightly shorter and thus interactions with SUR1 less probable. In previous papers, it was postulated that N-ter is a part of the opening-closing control mechanism.^10,21,25^ Point mutations preventing the helix formation in the proximal region of N-ter (D20–Q30), for example, by insertion of Pro residues, should result in modified conductivity of KATP. This observation, in turn, could be linked with genetic diseases related to glucose metabolism.

The data on IDR in KATP may be also useful in studies on the formation of such octameric potassium channels.^50^ Synthesized Kir6.2 and SUR1 subunits must be delivered to a membrane. Transport across a cell takes place. In cells, regions with condensates not embraced by membranes are observed. The liquid–liquid phase separation (LLPS) process may be involved in KATP assembly. Recently, the role of IDR in LLPS has been recognized.^52^ We hypothesize that our data on IDRs in KATP may facilitate interpretation of future experimental studies of ion channel formation scenarios.

## 3 Conclusions

The role of IDRs of proteins in living organisms is becoming better understood. Considering a large number of such regions recognized so far,^1,53^ both cytosolic and membrane, we are probably only at the beginning of this route. In the case of the KATP channel, vital for the proper functioning of the human body, our studies allow us to unveil the possible functional roles of yet unexplored fragments.

First of all, these regions may participate in “transferring information” between domains via the modulation of subunit–subunit interactions. In particular, this refers to the N-ter Kir6.2 tail, which we described in our recent paper,^25^ and the L0-loop described here. These fragments serve as links between SUR1, being an ATP intracellular concentration sensor, and the channel pore formed by Kir6.2 four subunits. Probably concerted motions in these two pathways lead to correct functioning of the complex.

Moreover, we show computationally that IDRs are involved in physiological ligand binding. The L0-loop, particularly the ABLOS part, contributes to the binding of both ATP and PIP2. The 900-loop, on the other hand, through the existence of a very rare glutamic acid-rich fragment, directly affects the MgADP binding site in NBD1 SUR1. Such stabilizing interaction prevents the excessive ligand fluctuations, which might otherwise lead to the ligand dissociation during spontaneous NBD domain relaxation.

Additionally, our study shows that the transient direct interactions between adjacent SUR1 units are possible through the 600-loops and 900-loops. Such contacts may modulate the stability of the characteristic propeller structure of the KATP channel. Due to location of the 600-loop at the close proximity to the CTD domain of Kir6.2, its high flexibility may be also functional—it may be a part of hypothetical of molecular bearing type Kir6.2/SUR1 cytosol located interface. Significance of these findings has yet to be confirmed by further both computational and experimental investigations.

## 4 Methods

### 4.1 System Preparation

The fully solvated KATP complex model was built based on the CryoEM structure of the human KATP in the activated form (PDB ID: 6C3P).^19^ Each Kir6.2 and SUR1 part was examined for missing side chains and loops using Schrodinger software. The minor missing regions (intra and extracellular loops of SUR1 shorter than 20 aa) were filled in using Prime-Schrodinger module, whereas two major missing regions in SUR1 (600-loop, and 900-loop, see Figure 1c) were modeled using the I-TASSER structure prediction server.^54,55^ The structure of the missing terminal parts of Kir6.2 subunits were predicted using the QUARK server.^56^ The newly added fragments were pre-equilibrated as described in Walczewska-Szewc and Nowak, 2020.^25^

Using CHARMM-GUI server,^57−59^ the model of KATP was embedded into an explicit palmitoyloleoyl-phosphatidylcholine bilayer with 10% phosphatidylinositol 4,5-bisphosphate (PIP2) in the inner leaflet of the membrane.^59^ Two MgADP molecules (with force–field parameters generated by CHARMM-GUI) were placed in consensus and degenerate binding sites in each SUR1. For the APO models, the binding sites remained unoccupied. The entire protein–membrane systems were solvated with the TIP3P water and 150 mM KCl, resulting in a simulation box 250 × 250 × 210 Å^3^ and consisted of approximately 1.3 mln atoms.

### 4.2 MD Simulations

All MD simulations were performed with GROMACS 2019.^60^ Bonds to hydrogen atoms were held rigid using LINCS algorithms,^61^ allowing us to use 2 fs time step. Periodic boundary conditions were used. Long-range electrostatic forces were calculated using the particle mesh Ewald method.^62^ The simulations were performed with a constant temperature of 310 K maintained using Nose–Hoover thermostat^63^ and a constant pressure of 1 atm (Parrinello–Rahman algorithm^64^). The CHARMM36m force field^65,66^ was used in all the MD simulations. Because our system combines both compact and disordered regions, the force field must be carefully chosen.^67^ The CHARMM36m force field used in this study has improved accuracy in generating conformational ensembles for IDR.^65^ The modified TIP3P water model intends to fix the problem of over-compactness by increasing the dispersion interactions between the protein and water.^65^ Although obtained IDR trajectories are still slightly more compact compared to experimental data,^68^ recent force field benchmarking studies^69,70^ show that CHARMM36m gives acceptable results when modeling systems containing both IDR and folded parts. After minimization and equilibration period (20 ns), 10 replicas of the system were run. Each simulation gave us a 500 ns trajectory. Two additional 500 ns simulations were run for the APO model.

### 4.3 Data Analysis

The structure assignment for each residue and time was calculated using Gromacs *do_dssp* tool. After reading a trajectory file, it computes the secondary structure for each time frame calling the *dssp* program.^71^ The PONDR score for each region was calculated using the Predictor of Natural Disordered Regions server (pondr.com).

Frequency of a ligand-residue and residue–residue contacts were calculated using the residue–residue contact score^72^ for each frame across the trajectory, setting the 0.1 threshold. rmsd, RMSF, PCA, and all structural properties of the system were calculated using Python scripts that utilize MDAnalysis, MDTraj, NumPy, SciPy, scikit-learn, and Matplotlib libraries. Couplings among parts of PREP in each system were analyzed using the dynamic cross-correlation matrices.^73^ VMD 1.9.3 was used for molecular structure visualization.^74^

## Data and Software Availability

All input files can be found on our GitHub repository: https://github.com/kszewc/IDR. MD trajectories are available on request—please contact me by e-mail specifying the range of data you are interested in.

## Supporting Information Available

Structural differences between the initial configuration based on 6C3P and after 500ns of MD simulation, detailed PCA and rmsd analysis of IDRs, interactions of the ED domain with ADP and the “anchor” residues, correlation matrices, and PIP_2_ occupancy (PDF)

## References

[ref1] Uversky V. N. Intrinsically Disordered Proteins and Their “Mysterious” (Meta)Physics. Front. Phys. 2019, 7, 10 10.3389/fphy.2019.00010.

[ref2] Lambrughi M.; Maiani E.; Aykac Fas B.; Shaw G. S.; Kragelund B. B.; Lindorff-Larsen K.; Teilum K.; Invernizzi G.; Papaleo E. Ubiquitin Interacting Motifs: Duality Between Structured and Disordered Motifs. Front. Mol. Biosci. 2021, 8, 676235 10.3389/fmolb.2021.676235. 34262938 PMC8273247

[ref3] Hibino H.; Inanobe A.; Furutani K.; Murakami S.; Findlay I.; Kurachi Y. Inwardly Rectifying Potassium Channels: Their Structure, Function, and Physiological Roles. Physiol. Rev. 2010, 90, 291–366. 10.1152/physrev.00021.2009. 20086079

[ref4] Ashcroft F. M. ATP-sensitive potassium channelopathies: focus on insulin secretion. J. Clin. Invest. 2005, 115, 2047–2058. 10.1172/jci25495. 16075046 PMC1180549

[ref5] Dunne M. J.; Cosgrove K. E.; Shepherd R. M.; Aynsley-green A.; Lindley K. J. Hyperinsulinism in Infancy: From Basic Science to Clinical Disease. Physiol. Rev. 2004, 84, 239–275. 10.1152/physrev.00022.2003. 14715916

[ref6] Kjaergaard M.; Kragelund B. B. Functions of intrinsic disorder in transmembrane proteins. Cell. Mol. Life Sci. 2017, 74, 3205–3224. 10.1007/s00018-017-2562-5. 28601983 PMC11107515

[ref7] Goretzki B.; Guhl C.; Tebbe F.; Harder J.-M.; Hellmich U. A. Unstructural Biology of TRP Ion Channels: The Role of Intrinsically Disordered Regions in Channel Function and Regulation. J. Mol. Biol. 2021, 433, 166931 10.1016/j.jmb.2021.166931. 33741410

[ref8] Sung M. W.; Driggers C. M.; Mostofian B.; Russo J. D.; Patton B. L.; Zuckerman D. M.; Shyng S.-L. Ligand-mediated Structural Dynamics of a Mammalian Pancreatic KATP Channel. J. Mol. Biol. 2022, 434, 167789 10.1016/j.jmb.2022.167789. 35964676 PMC9618280

[ref9] Ding D.; Wang M.; Wu J. X.; Kang Y.; Chen L. The Structural Basis for the Binding of Repaglinide to the Pancreatic KATP Channel. Cell Rep. 2019, 27, 1848–1857. 10.1016/j.celrep.2019.04.050. 31067468

[ref10] Martin G. M.; Sung M. W.; Yang Z.; Innes L. M.; Kandasamy B.; David L. L.; Yoshioka C.; Shyng S.-L. Mechanism of pharmacochaperoning in a mammalian KATP channel revealed by cryo-EM. eLife 2019, 8, e46417 10.7554/eLife.46417. 31343405 PMC6699824

[ref11] Sung M. W.; Yang Z.; Driggers C. M.; Patton B. L.; Mostofian B.; Russo J. D.; Zuckerman D. M.; Shyng S.-L. Vascular KATP channel structural dynamics reveal regulatory mechanism by Mg-nucleotides. Proc. Natl. Acad. Sci. U.S.A. 2021, 118, e2109441118 10.1073/pnas.2109441118. 34711681 PMC8694068

[ref12] Kikhney A. G.; Svergun D. I. A practical guide to small angle X-ray scattering (SAXS) of flexible and intrinsically disordered proteins. FEBS Lett. 2015, 589, 2570–2577. 10.1016/j.febslet.2015.08.027. 26320411

[ref13] Jensen M. R.; Zweckstetter M.; Huang J.-r.; Blackledge M. Exploring free-energy landscapes of intrinsically disordered proteins at atomic resolution using NMR spectroscopy. Chem. Rev. 2014, 114, 6632–6660. 10.1021/cr400688u. 24725176

[ref14] Brucale M.; Schuler B.; Samorì B. Single-molecule studies of intrinsically disordered proteins. Chem. Rev. 2014, 114, 3281–3317. 10.1021/cr400297g. 24432838

[ref15] Cukier R. I. Generating Intrinsically Disordered Protein Conformational Ensembles from a Database of Ramachandran Space Pair Residue Probabilities Using a Markov Chain. J. Phys. Chem. B 2018, 122, 9087–9101. 10.1021/acs.jpcb.8b05797. 30204435

[ref16] Shapovalov M. V.; Dunbrack R. L. Jr. A smoothed backbone-dependent rotamer library for proteins derived from adaptive kernel density estimates and regressions. Structure 2011, 19, 844–858. 10.1016/j.str.2011.03.019. 21645855 PMC3118414

[ref17] Ozenne V.; Bauer F.; Salmon L.; Huang J.-r.; Jensen M. R.; Segard S.; Bernadó P.; Charavay C.; Blackledge M. Flexible-meccano: a tool for the generation of explicit ensemble descriptions of intrinsically disordered proteins and their associated experimental observables. Bioinformatics 2012, 28, 1463–1470. 10.1093/bioinformatics/bts172. 22613562

[ref18] Aittoniemi J.; Fotinou C.; Craig T. J.; de Wet H.; Proks P.; Ashcroft F. M. SUR1: a unique ATP-binding cassette protein that functions as an ion channel regulator. Philos. Trans. R. Soc., B 2009, 364, 257–267. 10.1098/rstb.2008.0142. PMC2674095 18990670

[ref19] Lee K. P. K.; Chen J.; MacKinnon R. Molecular structure of human KATP in complex with ATP and ADP. eLife 2017, 6, e32481 10.7554/eLife.32481. 29286281 PMC5790381

[ref20] Martin G. M.; Kandasamy B.; DiMaio F.; Yoshioka C.; Shyng S.-L. Anti-diabetic drug binding site in a mammalian KATP channel revealed by Cryo-EM. eLife 2017, 6, e31054 10.7554/eLife.31054. 29035201 PMC5655142

[ref21] Wu J. X.; Ding D.; Wang M.; Kang Y.; Zeng X.; Chen L. Ligand binding and conformational changes of SUR1 subunit in pancreatic ATP-sensitive potassium channels. Prot. Cell 2018, 9, 553–567. 10.1007/s13238-018-0530-y. PMC5966361 29594720

[ref22] Martin G. M.; Yoshioka C.; Rex E. A.; Fay J. F.; Xie Q.; Whorton M. R.; Chen J. Z.; Shyng S.-L. Cryo-EM structure of the ATP-sensitive potassium channel illuminates mechanisms of assembly and gating. elife 2017, 6, e24149 10.7554/eLife.24149. 28092267 PMC5344670

[ref23] Sooklal C. R.; López-Alonso J. P.; Papp N.; Kanelis V. Phosphorylation Alters the Residual Structure and Interactions of the Regulatory L1 Linker Connecting NBD1 to the Membrane-Bound Domain in SUR2B. Biochemistry 2018, 57, 6278–6292. 10.1021/acs.biochem.8b00503. 30273482

[ref24] Mittag T.; Forman-Kay J. D. Atomic-level characterization of disordered protein ensembles. Curr. Opin. Struct. Biol. 2007, 17, 3–14. 10.1016/j.sbi.2007.01.009. 17250999

[ref25] Walczewska-Szewc K.; Nowak W. Structural Determinants of Insulin Release: Disordered N-Terminal Tail of Kir6.2 Affects Potassium Channel Dynamics through Interactions with Sulfonylurea Binding Region in a SUR1 Partner. J. Phys. Chem. B 2020, 124, 6198–6211. 10.1021/acs.jpcb.0c02720. 32598150 PMC7467719

[ref26] Babenko A. P.; Gonzalez G.; Bryan J. The N-Terminus of KIR6.2 Limits Spontaneous Bursting and Modulates the ATP-Inhibition of KATPChannels. Biochem. Biophys. Res. Commun. 1999, 255, 231–238. 10.1006/bbrc.1999.0172. 10049691

[ref27] Wu J. X.; Ding D.; Wang M.; Chen L. Structural Insights into the Inhibitory Mechanism of Insulin Secretagogues on the Pancreatic ATP-Sensitive Potassium Channel. Biochemistry 2020, 59, 18–25. 10.1021/acs.biochem.9b00692. 31566370

[ref28] Pipatpolkai T.; Usher S.; Stansfeld P. J.; Ashcroft F. M. New insights into KATP channel gene mutations and neonatal diabetes mellitus. Nat. Rev. Endocrinol. 2020, 16, 378–393. 10.1038/s41574-020-0351-y. 32376986

[ref29] Walczewska-Szewc K.; Nowak W. Spacial models of malfunctioned protein complexes help to elucidate signal transduction critical for insulin release. Biosystems 2019, 177, 48 10.1016/j.biosystems.2018.11.001. 30395892

[ref30] Zerangue N.; Schwappach B.; Jan Y. N.; Jan L. Y. A New ER Trafficking Signal Regulates the Subunit Stoichiometry of Plasma Membrane KATP Channels. Neuron 1999, 22, 537–548. 10.1016/s0896-6273(00)80708-4. 10197533

[ref31] Tucker S. J.; Gribble F. M.; Zhao C.; Trapp S.; Ashcroft F. M. Truncation of Kir6.2 produces ATP-sensitive K+ channels in the absence of the sulphonylurea receptor. Nature 1997, 387, 179 10.1038/387179a0. 9144288

[ref32] Sang Y.; Yang W.; Yan J.; Wu Y. KCNJ11 gene mutation analysis on nine Chinese patients with type 1B diabetes diagnosed before 3 years of age. J. Pediatr. Endocrinol. Metab. 2014, 27, 519 10.1515/jpem-2013-0163. 24698822

[ref33] Sabusap C. M.; Joshi D.; Simhaev L.; Oliver K. E.; Senderowitz H.; van Willigen M.; Braakman I.; Rab A.; Sorscher E. J.; Hong J. S. The CFTR P67L variant reveals a key role for N-terminal lasso helices in channel folding, maturation, and pharmacologic rescue. J. Biol. Chem. 2021, 296, 100598 10.1016/j.jbc.2021.100598. 33781744 PMC8102917

[ref34] Bründl M.; Pellikan S.; Stary-Weinzinger A. Simulating PIP(2)-Induced Gating Transitions in Kir6.2 Channels. Front. Mol. Biosci. 2021, 8, 711975 10.3389/fmolb.2021.711975. 34447786 PMC8384051

[ref35] Puljung M.; Vedovato N.; Usher S.; Ashcroft F. Activation mechanism of ATP-sensitive K+ channels explored with real-time nucleotide binding. eLife 2019, 8, e41103 10.7554/eLife.41103. 30789344 PMC6400584

[ref36] Balamurugan K.; Kavitha B.; Yang Z.; Mohan V.; Radha V.; Shyng S.-L. Functional characterization of activating mutations in the sulfonylurea receptor 1 (ABCC8) causing neonatal diabetes mellitus in Asian Indian children. Pediatr. Diabetes 2019, 20, 397–407. 10.1111/pedi.12843. 30861254 PMC11423867

[ref37] Bickers S. C.; Sayewich J. S.; Kanelis V. Intrinsically disordered regions regulate the activities of ATP binding cassette transporters. Biochim. Biophys. Acta, Biomembr. 2020, 1862, 183202 10.1016/j.bbamem.2020.183202. 31972165

[ref38] Burton M. J.; Kapetanaki S. M.; Chernova T.; Jamieson A. G.; Dorlet P.; Santolini J.; Moody P. C. E.; Mitcheson J. S.; Davies N. W.; Schmid R.; Raven E. L.; Storey N. M. A heme-binding domain controls regulation of ATP-dependent potassium channels. Proc. Natl. Acad. Sci. U.S.A. 2016, 113, 3785–3790. 10.1073/pnas.1600211113. 27006498 PMC4833257

[ref39] Flanagan S. E.; Dung V. C.; Houghton J. A. L.; Franco E.; Ngoc C. T. B.; Damhuis A.; Ashcroft F. M.; Harries L. W.; Ellard S. An ABCC8 Nonsense Mutation Causing Neonatal Diabetes Through Altered Transcript Expression. J. Clini. Res. Pediatr. Endocrinol. 2017, 9, 260–264. 10.4274/jcrpe.4624. PMC5596808 28663158

[ref40] Uversky V. N. The alphabet of intrinsic disorder. Intrinsically Disord. Proteins 2013, 1, e24684 10.4161/idp.24684. 28516010 PMC5424795

[ref41] Driggers C. M.; Shyng S.-L. Mechanistic insights on KATP channel regulation from cryo-EM structures. J. Gen. Physiol. 2023, 155, e202113046 10.1085/jgp.202113046. 36441147 PMC9700523

[ref42] Koster J. C.; Kurata H. T.; Enkvetchakul D.; Nichols C. G. DEND mutation in Kir6.2 (KCNJ11) reveals a flexible N-terminal region critical for ATP-sensing of the KATP channel. Biophys. J. 2008, 95, 4689 10.1529/biophysj.108.138685. 18708460 PMC2576385

[ref43] Walczewska-Szewc K.; Nowak W. Photo-Switchable Sulfonylureas Binding to ATP-Sensitive Potassium Channel Reveal the Mechanism of Light-Controlled Insulin Release. J. Phys. Chem. B 2021, 125, 13111–13121. 10.1021/acs.jpcb.1c07292. 34825567 PMC8667036

[ref44] Li N.; Wu J. X.; Ding D.; Cheng J.; Gao N.; Chen L. Structure of a Pancreatic ATP-Sensitive Potassium Channel. Cell 2017, 168, 101–110. 10.1016/j.cell.2016.12.028. 28086082

[ref45] Zhao C.; MacKinnon R. Molecular structure of an open human KATP channel. Proc. Natl. Acad. Sci. U.S.A. 2021, 118, e2112267118 10.1073/pnas.2112267118. 34815345 PMC8640745

[ref46] de Araujo E. D.; Alvarez C. P.; López-Alonso J. P.; Sooklal C. R.; Stagljar M.; Kanelis V. Phosphorylation-dependent changes in nucleotide binding, conformation, and dynamics of the first nucleotide binding domain (NBD1) of the sulfonylurea receptor 2B (SUR2B). J. Biol. Chem. 2015, 290, 22699–22714. 10.1074/jbc.m114.636233. 26198630 PMC4566242

[ref47] David C. C.; Jacobs D. J. Principal component analysis: a method for determining the essential dynamics of proteins. Methods Mol. Biol. 2014, 1084, 193–226. 10.1007/978-1-62703-658-0_11. 24061923 PMC4676806

[ref48] Verkest C.; Schaefer I.; Nees T. A.; Wang N.; Jegelka J. M.; Taberner F. J.; Lechner S. G. Intrinsically disordered intracellular domains control key features of the mechanically-gated ion channel PIEZO2. Nat. Commun. 2022, 13, 1365 10.1038/s41467-022-28974-6. 35292651 PMC8924262

[ref49] Camacho-Zarco A. R.; Schnapka V.; Guseva S.; Abyzov A.; Adamski W.; Milles S.; Jensen M. R.; Zidek L.; Salvi N.; Blackledge M. NMR Provides Unique Insight into the Functional Dynamics and Interactions of Intrinsically Disordered Proteins. Chem. Rev. 2022, 122, 9331–9356. 10.1021/acs.chemrev.1c01023. 35446534 PMC9136928

[ref50] He W.; Li X.; Xue H.; Yang Y.; Mencius J.; Bai L.; Zhang J.; Xu J.; Wu B.; Xue Y.; Quan S. Insights into the client protein release mechanism of the ATP-independent chaperone Spy. Nat. Commun. 2022, 13, 2818 10.1038/s41467-022-30499-x. 35595811 PMC9122904

[ref51] Rauscher S.; Gapsys V.; Gajda M. J.; Zweckstetter M.; de Groot B. L.; Grubmüller H. Structural Ensembles of Intrinsically Disordered Proteins Depend Strongly on Force Field: A Comparison to Experiment. J. Chem. Theor. Comput. 2015, 11, 5513–5524. 10.1021/acs.jctc.5b00736. 26574339

[ref52] Abyzov A.; Blackledge M.; Zweckstetter M. Conformational Dynamics of Intrinsically Disordered Proteins Regulate Biomolecular Condensate Chemistry. Chem. Rev. 2022, 122, 6719–6748. 10.1021/acs.chemrev.1c00774. 35179885 PMC8949871

[ref53] Wilson C. J.; Choy W.-Y.; Karttunen M. AlphaFold2: A Role for Disordered Protein/Region Prediction?. Int. J. Mol. Sci. 2022, 23, 4591 10.3390/ijms23094591. 35562983 PMC9104326

[ref54] Yang J.; Yan R.; Roy A.; Xu D.; Poisson J.; Zhang Y. The I-TASSER Suite: protein structure and function prediction. Nat. Methods 2015, 12, 7–8. 10.1038/nmeth.3213. PMC4428668 25549265

[ref55] Zhang Y. I-TASSER server for protein 3D structure prediction. BMC Bioinf. 2008, 9, 40 10.1186/1471-2105-9-40. PMC2245901 18215316

[ref56] Xu D.; Zhang Y. Ab initio protein structure assembly using continuous structure fragments and optimized knowledge-based force field. Proteins 2012, 80, 1715–1735. 10.1002/prot.24065. 22411565 PMC3370074

[ref57] Jo S.; Kim T.; Iyer V. G.; Im W. CHARMM-GUI: A web-based graphical user interface for CHARMM. J. Comput. Chem. 2008, 29, 1859–1865. 10.1002/jcc.20945. 18351591

[ref58] Lee J.; Cheng X.; Swails J. M.; Yeom M. S.; Eastman P. K.; Lemkul J. A.; Wei S.; Buckner J.; Jeong J. C.; Qi Y.; Jo S.; Pande V. S.; Case D. A.; Brooks C. L.; MacKerell A. D.; Klauda J. B.; Im W. CHARMM-GUI Input Generator for NAMD, GROMACS, AMBER, OpenMM, and CHARMM/OpenMM Simulations Using the CHARMM36 Additive Force Field. J. Chem. Theor. Comput. 2016, 12, 405–413. 10.1021/acs.jctc.5b00935. PMC4712441 26631602

[ref59] Wu E. L.; Cheng X.; Jo S.; Rui H.; Song K. C.; Dávila-Contreras E. M.; Qi Y.; Lee J.; Monje-Galvan V.; Venable R. M.; Klauda J. B.; Im W. CHARMM-GUIMembrane Buildertoward realistic biological membrane simulations. J. Comput. Chem. 2014, 35, 1997–2004. 10.1002/jcc.23702. 25130509 PMC4165794

[ref60] Abraham M. J.; Murtola T.; Schulz R.; Páll S.; Smith J. C.; Hess B.; Lindahl E. GROMACS: High performance molecular simulations through multi-level parallelism from laptops to supercomputers. SoftwareX 2015, 1–2, 19–25. 10.1016/j.softx.2015.06.001.

[ref61] Hess B. P-LINCS: A Parallel Linear Constraint Solver for Molecular Simulation. J. Chem. Theor. Comput. 2008, 4, 116 10.1021/ct700200b. 26619985

[ref62] Darden T.; York D.; Pedersen L. Particle mesh Ewald: AnN·log(N) method for Ewald sums in large systems. J. Chem. Phys. 1993, 98, 10089–10092. 10.1063/1.464397.

[ref63] Evans D. J.; Holian B. L. The Nose-Hoover thermostat. J. Chem. Phys. 1985, 83, 4069–4074. 10.1063/1.449071.

[ref64] Parrinello M.; Rahman A. Polymorphic transitions in single crystals: A new molecular dynamics method. J. Appl. Phys. 1981, 52, 7182–7190. 10.1063/1.328693.

[ref65] Huang J.; Rauscher S.; Nawrocki G.; Ran T.; Feig M.; de Groot B. L.; Grubmüller H.; MacKerell A. D. CHARMM36m: an improved force field for folded and intrinsically disordered proteins. Nat. Methods 2017, 14, 71–73. 10.1038/nmeth.4067. 27819658 PMC5199616

[ref66] Huang J.; MacKerell A. D. Jr. CHARMM36 all-atom additive protein force field: validation based on comparison to NMR data. J. Comput. Chem. 2013, 34, 2135–2145. 10.1002/jcc.23354. 23832629 PMC3800559

[ref67] Nerenberg P. S.; Jo B.; So C.; Tripathy A.; Head-Gordon T. Optimizing Solute-Water van der Waals Interactions To Reproduce Solvation Free Energies. J. Phys. Chem. B 2012, 116, 4524–4534. 10.1021/jp2118373. 22443635

[ref68] Robustelli P.; Piana S.; Shaw D. E. Developing a molecular dynamics force field for both folded and disordered protein states. Proc. Natl. Acad. Sci. U.S.A. 2018, 115, E4758–E4766. 10.1073/pnas.1800690115. 29735687 PMC6003505

[ref69] Samantray S.; Yin F.; Kav B.; Strodel B. Different Force Fields Give Rise to Different Amyloid Aggregation Pathways in Molecular Dynamics Simulations. J. Chem. Inf. Model. 2020, 60, 6462–6475. 10.1021/acs.jcim.0c01063. 33174726

[ref70] Mu J.; Liu H.; Zhang J.; Luo R.; Chen H.-F. Recent Force Field Strategies for Intrinsically Disordered Proteins. J. Chem. Inf. Model. 2021, 61, 1037–1047. 10.1021/acs.jcim.0c01175. 33591749 PMC8256680

[ref71] Kabsch W.; Sander C. Dictionary of protein secondary structure: Pattern recognition of hydrogen-bonded and geometrical features. Biopolymers 1983, 22, 2577–2637. 10.1002/bip.360221211. 6667333

[ref72] Zhou Q.; Yang D.; Wu M.; Guo Y.; Guo W.; Zhong L.; Cai X.; Dai A.; Jang W.; Shakhnovich E. I.; Liu Z.-J.; Stevens R. C.; Lambert N. A.; Babu M. M.; Wang M.-W.; Zhao S. Common activation mechanism of class A GPCRs. eLife 2019, 8, e50279 10.7554/eLife.50279. 31855179 PMC6954041

[ref73] Kasahara K.; Fukuda I.; Nakamura H. A Novel Approach of Dynamic Cross Correlation Analysis on Molecular Dynamics Simulations and Its Application to Ets1 Dimer-DNA Complex. PLoS One 2014, 9, e112419 10.1371/journal.pone.0112419. 25380315 PMC4224484

[ref74] Humphrey W.; Dalke A.; Schulten K. VMD: Visual molecular dynamics. J. Mol. Graph. 1996, 14, 33–38. 10.1016/0263-7855(96)00018-5. 8744570

