# Supplementary material for: Structural Insights into ATP-Sensitive Potassium Channel Mechanics: A Role of Intrinsically Disordered Regions
Source: J Chem Inf Model. 2023 Feb 6;63(6):1806–18. doi: 10.1021/acs.jcim.2c01196 (PMC10052335; doi:10.1021/acs.jcim.2c01196)
Supplement: Supplementary file 1 — ci2c01196_si_001.pdf [file ci2c01196_si_001.pdf]

SUPPORTING INFORMATION:

Structural Insights into ATP-Sensitive Potassium Channel Mechanics: a Role of Intrinsically Disordered Regions.

Katarzyna Walczewska-Szewc*, Wiesław Nowak

Institute of Physics, Faculty of Physics, Astronomy and Informatics, Nicolaus Copernicus University in Toruń, ul. Grudziądzka 5, 87-100 Toruń, Poland. * kszewc@umk.pl

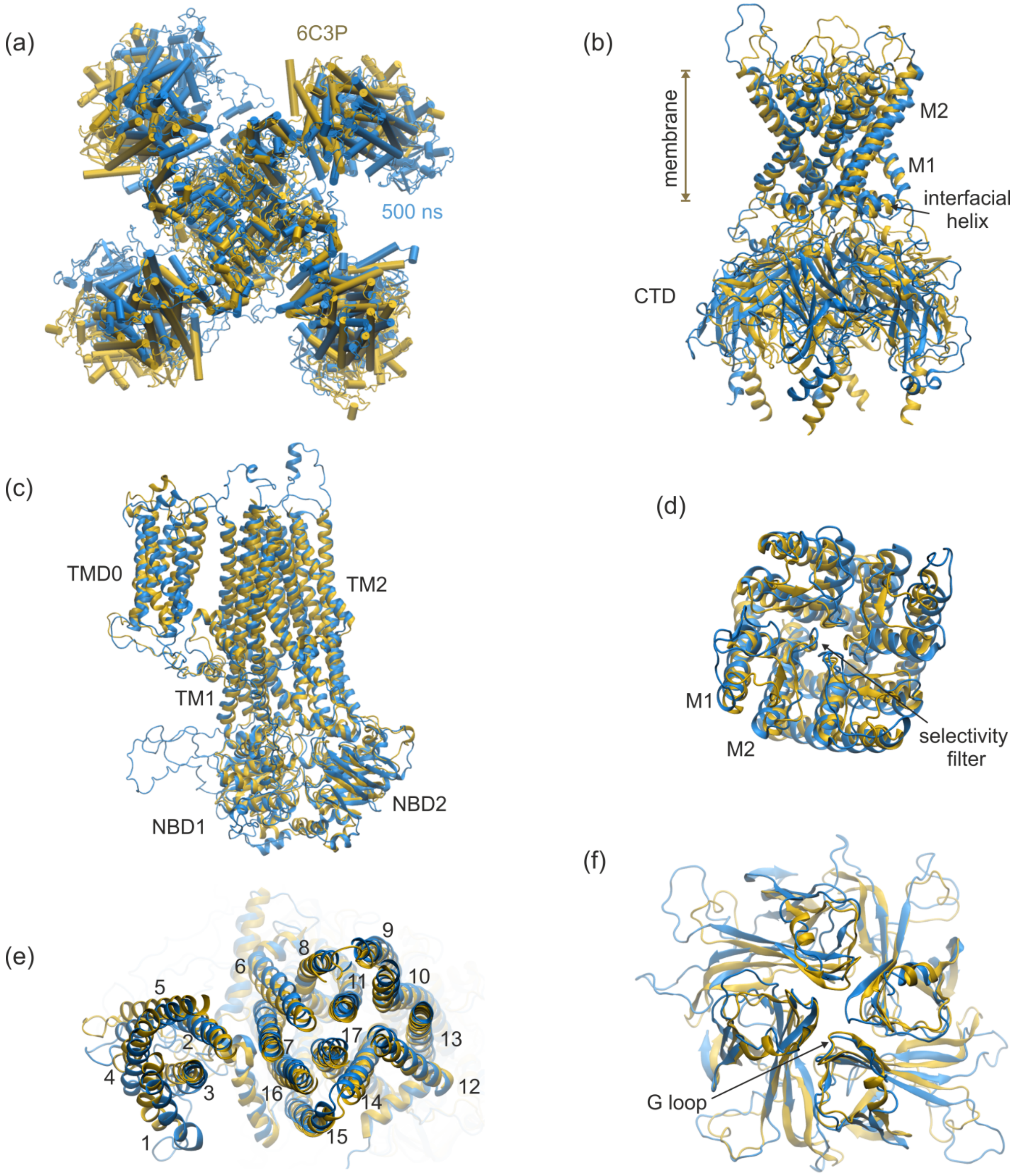

Figure S1. Structural differences between the initial configuration based on 6C3P and after 500ns of MD simulation. Whole channel (a); Kir6.2 tetramer-side view (b); SUR1-side view (c); pore formed by transmembrane part of Kir6.2 (d); transmembrane part of SUR1 (e); CTD part of Kir6.2 tetramer (f).

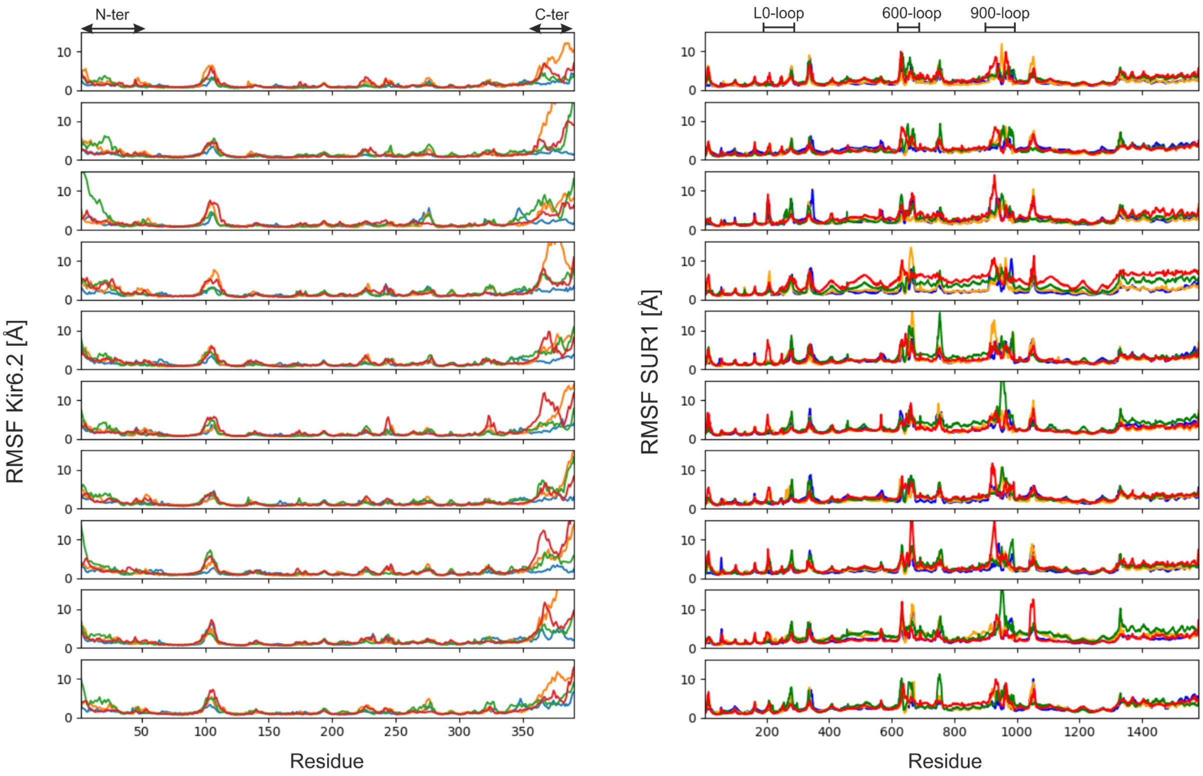

Figure S2. Root mean square fluctuations for Kir6.2 (a) and SUR1 (b) subunits of KATP calculated for each simulation run (separate panels) and each of four chains (separate colors).

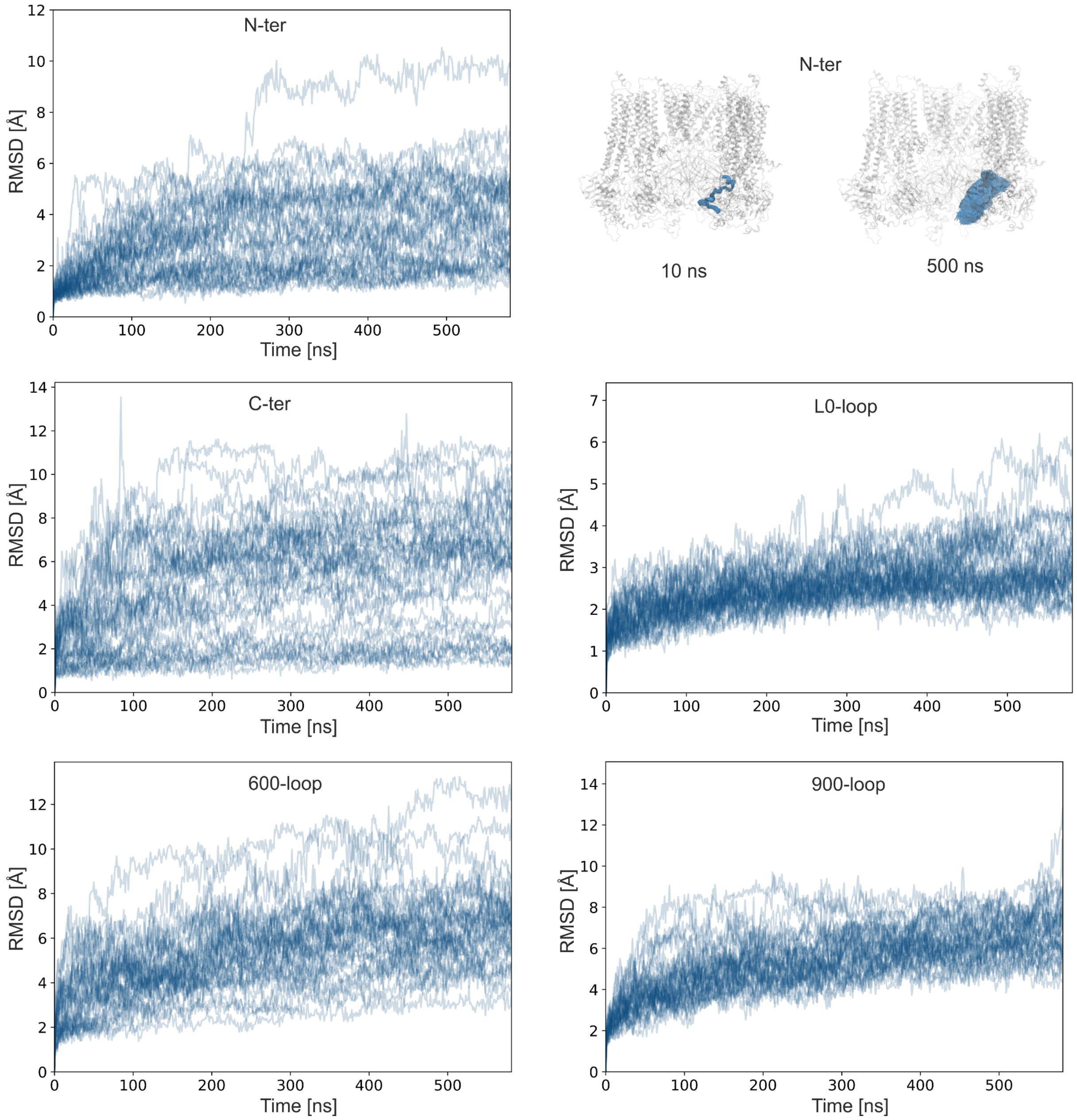

Figure S3. Cα root mean square deviation (RMSD) of disordered parts throughout simulation calculated separately for each run and each protein chain (10 runs x 4 chains). The structures in the upper left corner show the increase of the volume sampled by N-ter from 10 to 500 ns of simulation.

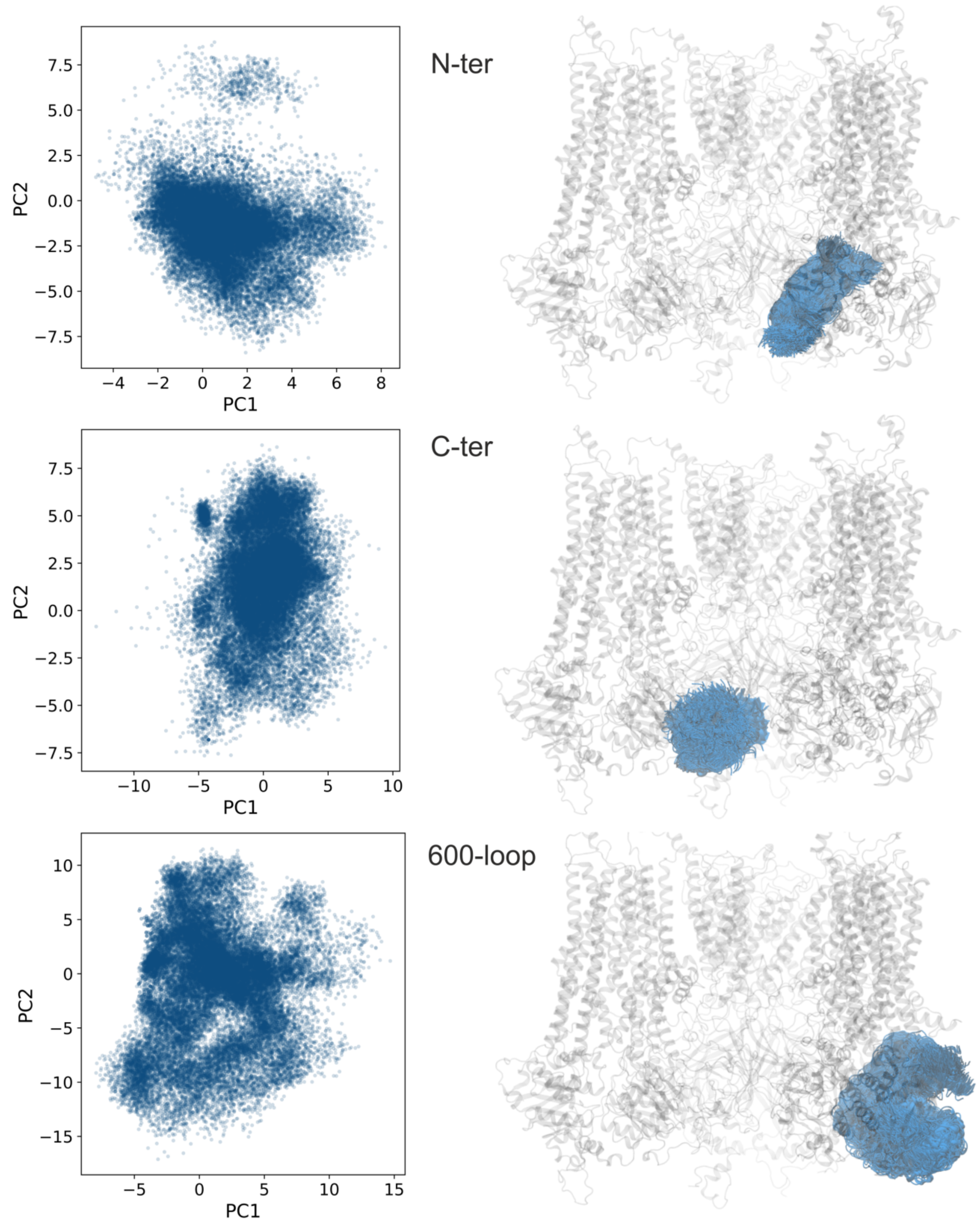

Figure S4. The principal components analysis of IDRs dynamics (left) and the volumes sampled by IDRs throughout 500ns simulation (right).

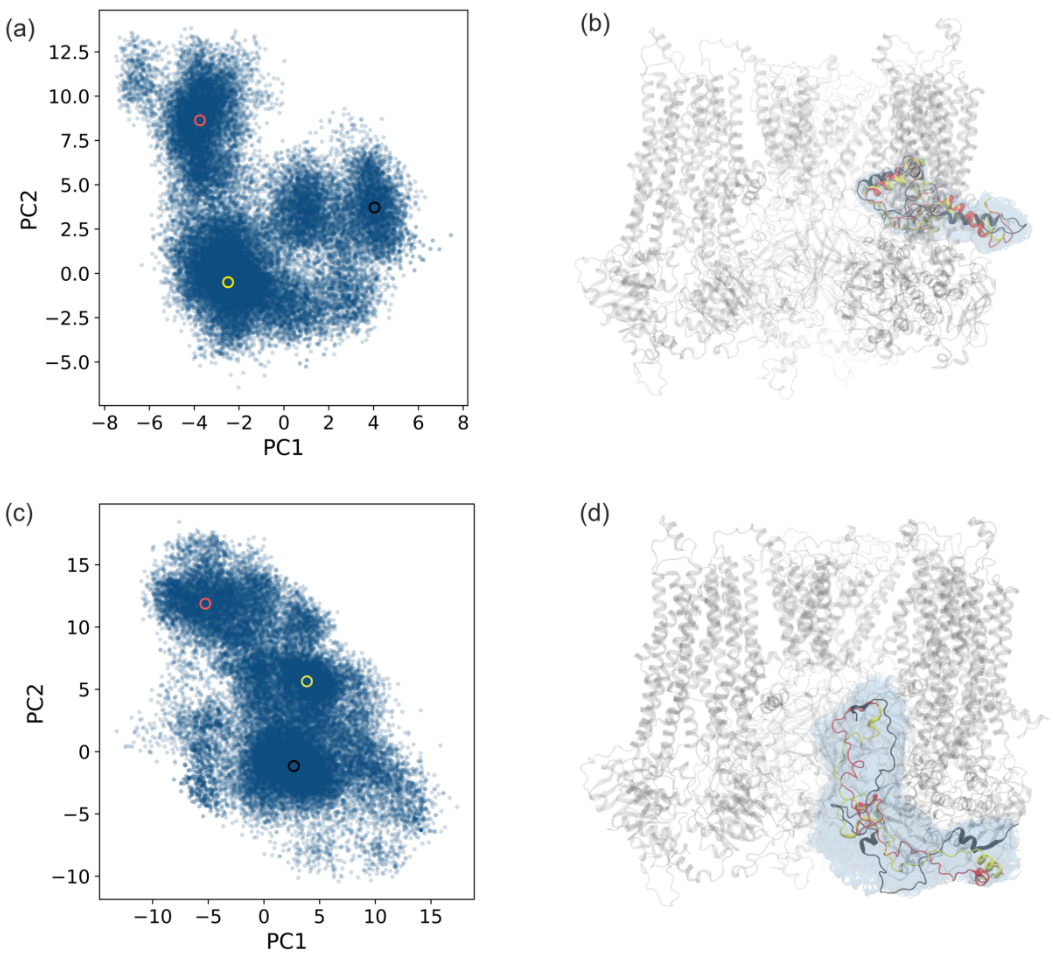

Figure S5. The principl components analysis of IDRs dynamics of L0-loop and 900-loop (a,c). Red, black and yellow circles denote the centers of three distinctive conformational clusters. The conformation corresponding to each cluster center is shown in the right panel (b,d), together with the volumes sampled by IDRs throughout 500ns simulation.

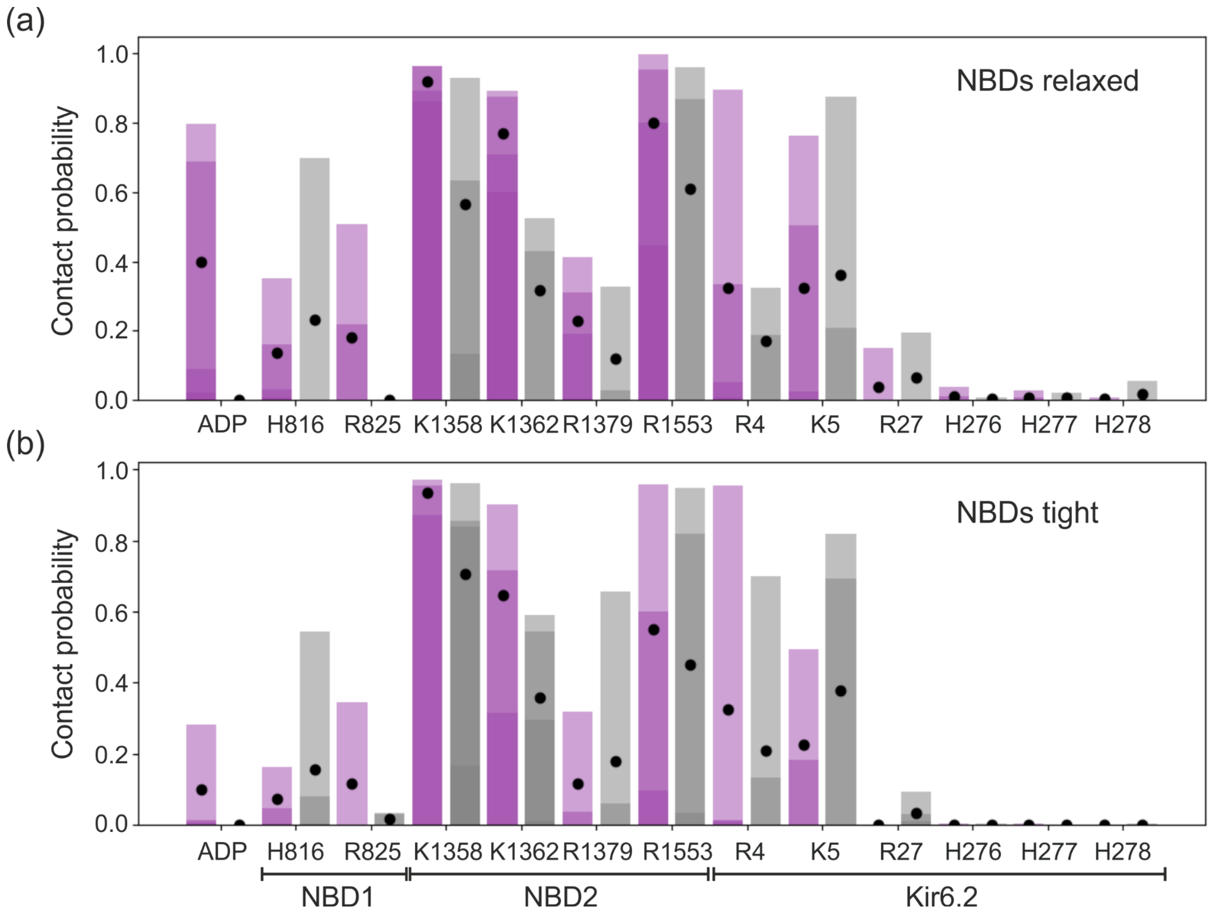

Figure S6. Interactions of the ED domain with ADP and the "anchor" residues of NBDs of SUR1 and Kir6.2. Slight relaxation of the domains changes the probability of close contact between residues.

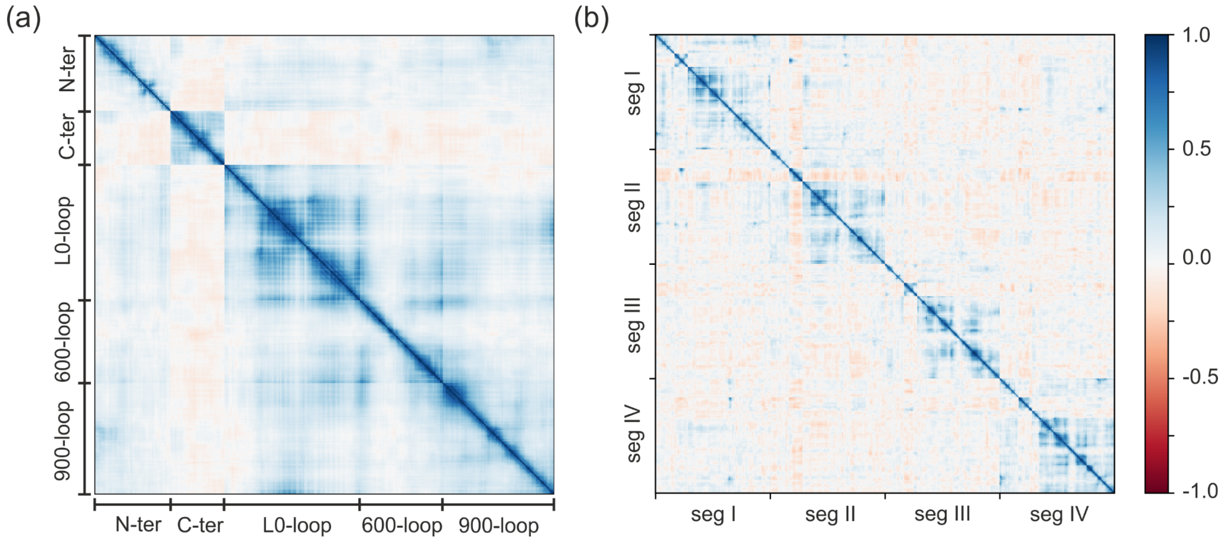

Figure S7. Correlation matrices calculated for each IDR from 10 x 500 ns x four chains MD trajectories data (a) and for IDRs from separate chains (10 x 500 ns MD data).

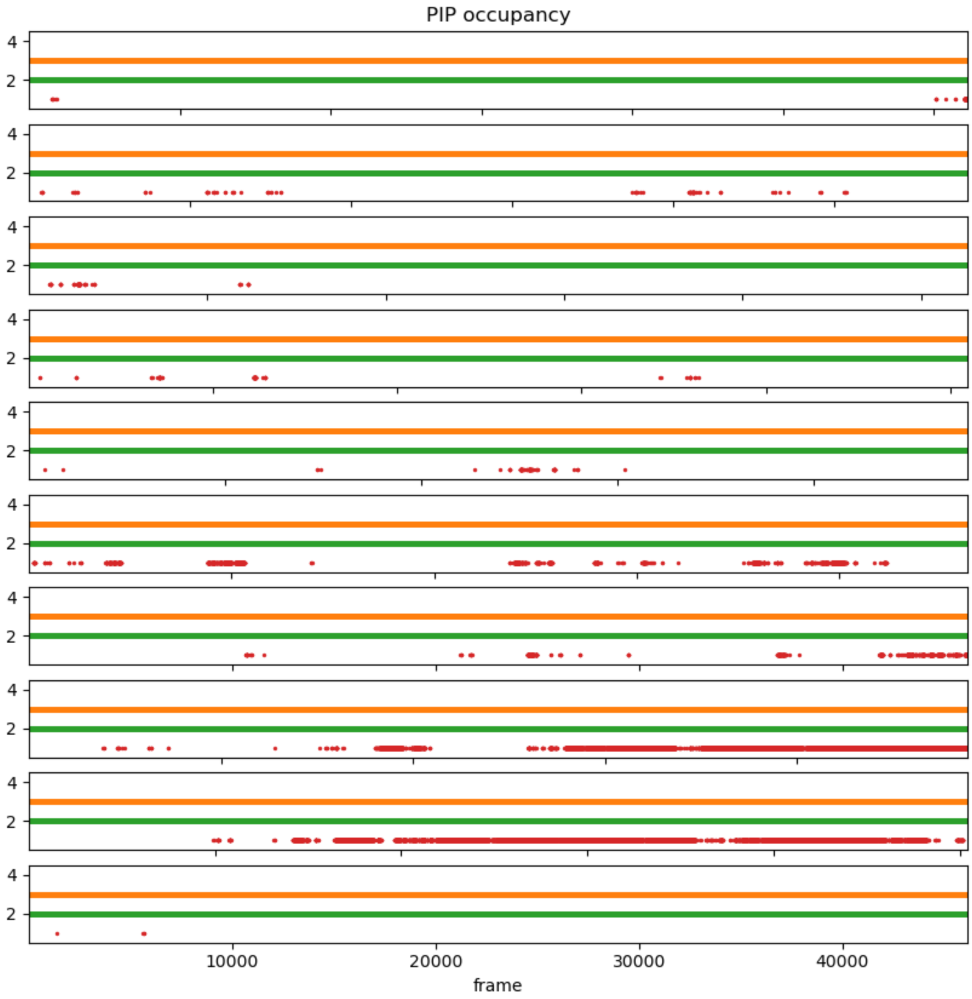

Figure S8 PIP_2 occupancy in the Kir6.2 binding site in all MD repeats.
